# Gene-x-environment analysis supports protective effects of eveningness chronotype on self-reported and actigraphy-derived sleep duration among those who always work night shifts in the UK Biobank

**DOI:** 10.1093/sleep/zsad023

**Published:** 2023-02-06

**Authors:** Evelina T Akimova, Riley Taiji, Xuejie Ding, Melinda C Mills

**Affiliations:** Leverhulme Centre for Demographic Science, University of Oxford, Oxford, UK; Nuffield College, University of Oxford, Oxford, UK; Leverhulme Centre for Demographic Science, University of Oxford, Oxford, UK; Nuffield College, University of Oxford, Oxford, UK; Leverhulme Centre for Demographic Science, University of Oxford, Oxford, UK; Nuffield College, University of Oxford, Oxford, UK; Leverhulme Centre for Demographic Science, University of Oxford, Oxford, UK; Nuffield College, University of Oxford, Oxford, UK; Department of Economics, Econometrics and Finance, University of Groningen, Groningen, The Netherlands; Department of Genetics, University Medical Centre Groningen, Groningen, The Netherlands

**Keywords:** night shift work, sleep duration, eveningness chronotype, GWAS, gene-environment interaction

## Abstract

Previous research has linked having an eveningness chronotype with a higher tolerance for night shift work, suggesting the ability to work nights without health consequences may partially depend upon having a circadian clock optimized for these times. As chronotypes entrain over time to environmental cues, it remains unclear whether higher relative eveningness among healthy night workers reflects a moderating or mediating effect of chronotype on health. We address these concerns conducting a genome-wide association study and utilizing a polygenic score (PGS) for eveningness as a time-invariant measure of chronotype. On a sample of 53 211 workers in the UK Biobank (2006–2018), we focus on the effects of night shift work on sleep duration, a channel through which night shift work adversely affects health. We ask whether a higher predisposition toward eveningness promotes night shift work tolerance. Results indicate that regular night shift work is associated with a 13-minute (3.5%) reduction in self-reported sleep per night relative to those who never work these hours (95% confidence interval [CI] = −17:01, −8:36). We find that eveningness has a strong protective effect on night workers: a one-SD increase in the PGS is associated with a 4-minute (28%) reduction in the night shift work sleep penalty per night (CI = 0:10, 7:04). This protective effect is pronounced for those working the longest hours. Consistent patterns are observed with an actigraphy-derived measure of sleep duration. These findings indicate that solutions to health consequences of night shift work should take individual differences in chronotype into account.

## Graphical Abstract

**Graphical Abstract:**
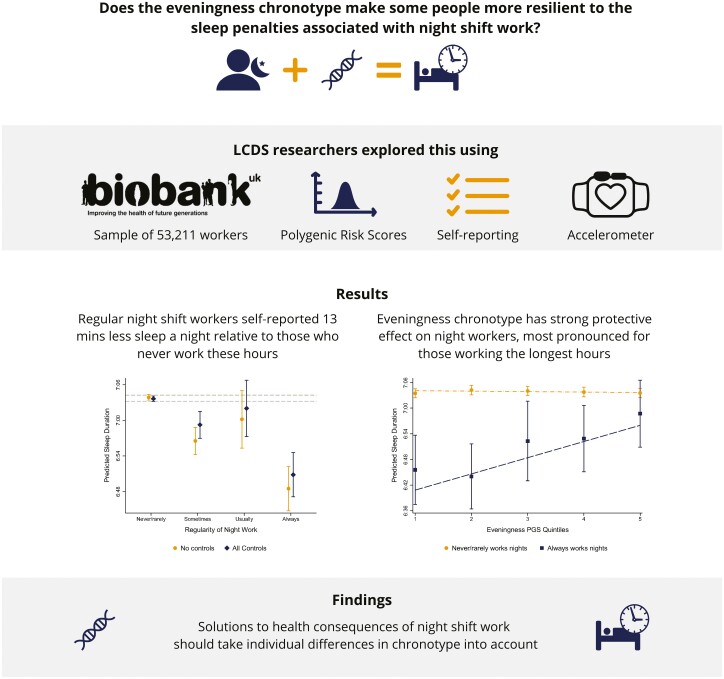

**Statement of Significance**

Night shift employees represent up to one-quarter of working populations. Increasing evidence shows that night shift work is a risk factor for various health conditions. Prolonged circadian disruption is one mechanism driving adverse effects. Individual differences in chronotypes, however, introduce differences in their abilities to adapt and tolerate shift schedules. This article examines whether genetic propensity for eveningness protects night workers against sleep penalties. Using data from the UK Biobank and multiple genetic, self-reported and accelerometer measures, we found evidence of the protective effect of a genetic propensity for eveningness, strongest for those working longer night shifts. Interventions to alleviate negative health consequences of night work should take individual differences of chronotype into account.

## Introduction

Between 15% and 20% of the US working population and 11% and 25% of UK employees in the public sector are engaged in some form of night shift employment [1, 2]. Night shift work is defined as work that typically occurs between the hours of 10:00 pm and 6:00 am. A growing body of literature has found that it represents a serious public health concern, including an increased risk of depression, coronary heart disease, type-2 diabetes, and functional gastrointestinal disorders (e.g. Foster *et al*. [3], Gu *et al*. [4], Lee *et al*. [5], Vetter *et al*. [6]). Links with various cancers have also been documented, with the World Health Organization recently labeling night work as a probable carcinogen [1, 7–9].

The likely mechanism of how night shift work adversely affects health is primarily through prolonged circadian disruption that results in subsequent reductions in sleep duration and quality. Circadian disruption results in immediate chronic sleep restriction, sleep-wake disturbances and are linked with shorter sleep duration and sleep disorders, common among shift workers [10]. Individuals working night shifts typically experience a misalignment between endogenous circadian rhythms and their actual sleep-wake cycle [11]. This “social jetlag” (i.e. the discordance between social and biological timing) [12] leads not only to the accumulation of sleep deficits over time, but also the dysregulation of the immune system as well as alterations in a wide range of physiological parameters, including glucose and insulin levels [13, 14]. In the short-term, circadian misalignment brought on by night shift work can manifest in what has been termed the “shift work sleep disorder,” characterized by persistent symptoms of fatigue, anxiety, and insomnia [15]. Our underlying assumption is that night shift work can result in sleep disruption and subsequently negative health outcomes, supported by considerable empirical evidence [16–20]. We do not claim that a longer sleep duration is associated with better health outcomes, since short (<7 h) and long (>9 h) sleep have both been associated with poor health [21]. Rather, our study focuses on circadian misalignment due to night shift work.

A potential protective factor against the side-effects of night shift work, however, is the timing of the endogenous circadian clock, or chronotype. Every individual possesses an endogenous circadian rhythm, which is the result of the interaction of their genetic makeup and environment, and, at least in part, dictates which times of day they function best [22]. Individuals with later rhythms—often referred to as “owls”—tend to do best with later bedtimes and rising times, while individuals with earlier rhythms—so-called, “larks”—tend to feel tired earlier in the evening and rise earlier. The majority of the population (~60%–80%) exists on a continuum somewhere between these poles [23, 24].

A wide body of literature has linked the eveningness chronotype with adaptation to and long-term tolerance of night shift work schedules, as evidenced by lower incidence of deleterious health outcomes including digestive problems, persistent fatigue, and sleep problems (e.g. Gamble *et al*. [25], Juda *et al*. [26], Saksvik *et al*.[27]). A recent study found that minimizing the discordance between work shift timing and chronotype—such that extreme morning-typed individuals avoid night work and vice versa (what the authors called “chronotype-adjusted shifts”)—significantly improves sleep duration and quality as well as overall well-being [28]. Such research broadly suggests that the ability to adapt to employment occurring during night hours may partially depend upon having a circadian clock optimized for these times.

A potential source of bias in these studies, however, has been the reliance on self-reported measures of chronotype drawn from cross-sectional data, with recent meta-reviews suggesting that longitudinal evidence is rare [27, 29]. A key property of circadian rhythms is their ability to be entrained over time to environmental cues (or *zeitgebers*), such as natural and synthetic light-dark cycles [22, 30]. This naturally raises concerns surrounding the direction of causality between night work, chronotype, and health outcomes. That is, it is difficult to determine whether higher rates of eveningness among the night workers who cope best is reflective of a moderating effect of chronotype or instead a mediating effect. Indeed, as Saksvik *et al*. [27] note, it may be that greater self-reported eveningness among healthy night shift workers simply reflects a form of adaptation—and thus a treatment response—rather than a true and stable trait.

In this study, we address these concerns by conducting a genome-wide association study (GWAS), producing and applying a polygenic score (PGS) for eveningness as a time-invariant measure of chronotype. A PGS is an index that linearly aggregates the estimated contributions of thousands of genetic variants to a trait of interest, using weights obtained from a GWAS. Utilizing 2006–2018 data from the UK Biobank (UKB), we focus on the effects of night work on sleep duration (posited as one of the central mechanisms through which night work exerts its adverse effects on health) and ask whether a higher genetic predisposition toward eveningness promotes greater night work tolerance.

## Methods

### Study population

We used cross-sectional data from the UKB collected between 2006 and 2018. The UKB provides genetic, demographic, and employment information on roughly 502 599 British respondents between the ages of 39 and 70. We restricted our analysis to individuals of White British ancestry who were genotyped and had levels of heterozygosity within ±3 SD’s from the mean (*n* = 455 538). We restricted analysis to White British ancestry individuals to avoid spurious genetic associations brought on by population stratification [31, 32]. The majority of existing genetic discoveries (GWASs) have taken place in European ancestry populations due to insufficient data for a large enough sample in other groups [33, 34], which our study also suffers from. PGSs derived from a GWAS in one ancestry group cannot be directly used for prediction in other groups due to differences such as Linkage Disequilibrium (LD), allele frequencies, and genetic architecture [33].

The application of a PGSs requires a nonoverlapping sample upon which to perform a GWAS [32]. To perform our primary GWAS of eveningness, we, therefore, selected 33.3% (*n* = 151 866) of the sample for use as the prediction set and reserved the remaining 66.7% (*n* = 303 672) as the reference set to run the GWAS on. We return to this latter analysis in subsequent discussions. We placed all dyads with a KING kinship coefficient ≥ 0.0884 (i.e. second-degree relations or closer) in the prediction set, thereby creating a reference set where all individuals were sufficiently unrelated. In the prediction set, we then created a family ID unique to every grouping of related individuals and cluster standard errors around this ID in all analyses (see Supplementary Appendix A.1). The analytic sample for this analysis included individuals who, at the time of data collection, were between the ages of 39–65 and in regular paid or self-employment of at least 10 h per week. On the prediction set, this amounted to a sample of 82 312. After removing individuals with missing values on our covariates of interest, a sample of 53 211 remained. For details on sample loss tied to each restriction, see Supplementary Appendix A.2. For details on analytic sample representativeness, see Supplementary Appendix A.3.

### Assessing sleep duration

The primary dependent variable for this study is sleep duration. We measure this using respondents’ self-reported sleep duration at the time of data collection, with sensitivity analyses measuring an actigraphy-derived measure of sleep duration. Respondents were asked, “About how many hours of sleep do you get in every 24 hours?” If respondents’ sleep duration varied a lot, they were asked to give the average time for a 24-hour day in the last 4 weeks. We modeled sleep duration as linear and looked at the change in minutes associated with night shift work. To minimize the effect of arbitrary variation in the upper and lower bounds, we capped sleep duration at between 3 and 12 hours/day.

We also conducted sensitivity analyses of sleep duration applying a more objective, actigraphy-derived measure of sleep duration. For these analyses, we use a subset of the prediction set that was provided wrist-worn accelerometers for 1 week (*n* = 30 530). We further restricted this group to those that were genotyped, in paid employment of at least 10 h per week and had non-missing values on our covariates of interest (*n* = 12 072). We described the construction of the actigraphy-derived sleep duration measure in Supplementary Appendix C.

### Assessing night shift work

The primary treatment variable for this study was the regularity with which a respondent reports working night shifts in their main job at the time of data collection. This measure was derived from a two-part question asked to individuals in paid or self-employment. Respondents were first asked whether their main job involved shift work, or “…a schedule that falls outside of the normal daytime hours.” Individuals who responded *sometimes*, *usually*, or *always* were then asked how often their “…work involve[s] night shifts.” Night shifts were defined in the UKB questionnaire as “...a work schedule that involves working through the normal sleeping hours, for instance working through the hours of 12:00 am and 6:00 am.” Possible response options were: (1) *never/rarely*, (2) *sometimes*, (3) *usually*, or (4) *always*. If a respondent had more than one job, they were advised to answer this question regarding their main job only.

Maintaining the original coding in the questionnaire, we modeled the regularity of night shift work as categorical, using those who *never/rarely* worked nights shifts as the reference group. Individuals who reported *never/rarely* working shifts in general in the first part of the question (*n* = 44 522) were also added to this reference group. A total of 2524 individuals reported *sometimes* working nights, 703 *usually* work nights, and 1295 *always* working nights. Frequencies for the recoded responses can be found in Table 1. Importantly, our analytic approach did not assume that the regularity of shift work is a continuous variable, but rather as a categorical one. This decision was based on the notion that the types of jobs in different shift work categories vary remarkably (Supplementary Appendix E and Tables S6–S8). Moreover, not only did occupations differ, but the occupations where sleep penalties were most pronounced were also distinct (Supplementary Appendix E and Figures S3–S5). Those who *always* engaged in night work, sales and white-collar workers had the most pronounced sleep deficits, while for those in *usual* night work, manufacturing and trades experience reaped the largest sleep deficits.

**Table 1.** Regularity of night work, by sex and work hours

	Total		Females		Males	
	*n*	Pct.	n	Pct.	n	Pct.
Regularity of night work						
Never/rarely	48 689	91.50%	26 065	93.95%	22 624	88.83%
Sometimes	2524	4.74%	949	3.42%	1575	6.18%
Usually	703	1.32%	259	0.93%	444	1.74%
Always	1295	2.43%	470	1.69%	825	3.24%
*N*	53 211		27 743		25 468	
	**Work hours**					
	**10–34 work hours/wk**		**35–44 work hours/wk**		**>44 work hours/wk**	
	***n***	**Pct.**	***n***	**Pct.**	***n***	**Pct.**
Regularity of night work						
Never/rarely	15 323	94.59%	23 013	91.40%	10 810	87.47%
Sometimes	470	3.00%	1156	4.59%	930	7.52%
Usually	130	0.83%	377	1.50%	207	1.67%
Always	248	1.58%	631	2.51%	412	3.33%
* N*	15 675		25 177		12 359	

Estimates are produced from a one-third random sample of the UK Biobank. The sample is further restricted to individuals between the ages of 39 and 65 who are in paid employment of at least 10 h per week.

### Assessing eveningness

The primary modifying variable of interest in this study was an individual’s genetic propensity for eveningness, which was measured using a polygenic risk score (PGS) derived from our GWAS. A PGS is an index that linearly aggregates the estimated effects of individual single nucleotide polymorphisms (SNPs) on a trait of interest, using weights derived from GWAS. The score can be thought of as a measure of an individual’s predisposition toward a given trait based on his or her genetic make-up. A PGS for individual *i* is defined as a weighted sum of that person’s genotypes at *J* loci. The score can be expressed as


$$\begin{array}{l} PGS_{i}=\;\underset{j=1}{\overset{J}{\sum }}\;x_{ij}w_{j} \end{array}$$

where *x* is individual *i*’s genotype (0, 1, 2) at variant *j* and *w* is a weight constructed from coefficients derived from a GWAS [32].

Our primary GWAS was performed on self-reported eveningness using a randomly selected 66.7% (*n* = 303 672) of the total UKB unrelated and genotyped sample, with the remaining 33.3% (*n* = 151 866) reserved as the prediction set. Self-reported eveningness was derived from a survey item asking respondents to report whether they, “…consider [themselves] to be”: (1) *definitely a morning person*, (2) *more a morning than evening person*, (3) *more an evening than a morning person*, (4) *definitely an evening person*, or (5) *do not know*. Following the approach used by Jones *et al*. [35, 36] in their chronotype GWAS, we coded these responses −2, −1, 1, 2, and 0, respectively.

Standard quality control (QC) thresholds were applied to the directly assayed and imputed UKB genetic data, resulting in a total of 5 666 911 autosomal SNPs upon which we ran the association study using Plink [37, 38]. For per individual QC, we removed individuals with a genetic relatedness greater than or equal to second degree (a King kinship coefficient ≥ 0.0884). We also removed individuals with heterozygosity >3 SD’s from the mean. For per SNP QC, the following thresholds were applied: (1) missing rate per SNP ≤ 0.05; (2) missing rate per person ≤ 0.03; (3) Hardy-Weinberg Equilibrium significance ≤ 0.00001; (4) minor allele frequency ≥ 0.01. In the GWAS, we modelled eveningness as a linear outcome and adjusted for age, sex, the first five genetic principal components, assessment centre (categorical), and a derived variable representing the genotyping release (categorical; UKBiLEVE array, UKB Axiom array interim release, and UKB Axiom array full release). The resultant summary statistics were then used to construct the PGS on the prediction set using PRSice [39]. A total of 340 381 variants were included after clumping at a radius of 250 kb and using a best-fit-inferred *p*-value threshold of 0.08. The final PGS performed reasonably well in predicting self-reported eveningness, with an *R*-squared of 0.021 (2%) and an *F*-statistic of 3290.31 (*p* < 0.001). The final score was standardized to a cross-sample mean of 0. More information on our GWAS and PGS calculation can be found in Supplementary Appendix B.

We also compared the results of our own GWAS with those available for similar chronotype phenotypes from Jones *et al*. [35, 36] by computing SNP-heritability and genetic overlap estimates using LD Score regression and LDSC software [40]. See Supplementary Appendix B.2, where we provide a detailed discussion of the methods and results. In summary, we found that our estimates were analytically identical to those from Jones *et al*. [35, 36], with the analyses provided elsewhere (see Supplementary Figure S2; genetic correlations are of −1 where a negative value is due reverse coding we employed to measure eveningness instead of morningness). We also observed an expected decrease in SNP-heritability due to our smaller sample size but it is not substantial (Supplementary Figure S1).

One important point is that this PGS still relied on the validity of the self-reported measure of eveningness used in the GWAS. As argued earlier, self-reports of chronotype as a measure of one’s true chronotype may be susceptible to nonrandom measurement error; patterns of socialization and—more importantly—the timing of one’s work schedule over time will likely affect how individuals self-identify their chronotype. However, biases relating to measurement error should be reduced in the present context since we instrument these self-reports with genotypic variation. As Lane *et al*. [41] argue, since individuals do not know their genotype, any phenotypic misclassification (i.e. misreported chronotype) will be random with respect to genotype. We return to the validity in subsequent discussions.

### Covariates

We adjusted for several potential confounding variables of the relationship between night work and sleep duration. Covariates were also included if they were posited to be directly related to sleep duration but only indirectly related to night work as such factors have been shown through simulations to reduce bias with sufficient sample size [42].

Demographic covariates included age group (40–45; 46–50; 51–55; 56–60; ≥61); sex; presence of child/grandchild in household (binary); presence of child/grandchild in household (binary); lives in urban area (binary); and years of education. To account for varying job demands, we adjusted for occupational class (SIOPS scale); work hours per week (10–36 h/wk; 37–45 h/wk; ≥46 h/wk); whether the job usually/always involves manual/heavy labor (binary); whether the job usually/always involved sitting/standing (binary); as well as total weekly commuting distance (30 miles or less/wk; 31–80 miles/wk; ≥81 miles/wk).

Along with this, we adjusted for the use of substances that affect sleep duration: regularity of alcohol use (never; special occasions; 1–3 times/month; 1–2 times/wk; 3–4 times/week; daily or almost daily); and current smoking (binary). We adjusted for a binary indicator of whether the respondent reported snoring as this has been shown to be associated with sleep duration as well as certain deleterious lifestyle factors [43]. We also adjusted for a 12-point neuroticism score, shown to both increase the likelihood of night shift work and reduce sleep duration. Lastly, when modeling the effects of the PGS for eveningness, we adjusted for the first five genetic principal components to account for confounding induced by population stratification. Descriptive statistics by the regularity of night work for all covariates are presented in Supplementary Appendix D.

### Analytical methods

In this study, our primary aims were to (1) estimate the sleep penalties associated with night shift work, and (2) identify whether these sleep penalties were modified by one’s genetic propensity for eveningness. To do so, we fit the following ordinary least squares model:


$$\begin{array}{l} SleepDuration=a+\;\beta_{1-3}NSW_{1-3}+\;\beta_{4}Eve+\\ \;\beta_{5-6}NSW_{1-3}*Eve+\;\beta_{7}Z+\;e \end{array}$$

where, $NSW_{1-3}$ refers to the three dummy variables for the regularity of night shift work (sometimes, usually, always; with never/rarely as the reference group), Everefers to the standardized PGS for eveningness, and Zrefers to a vector of covariates. β_1–3_ denotes the change in sleep duration associated with each respective regularity of night shift work, and β_5–6_ shows how this sleep change varies depending on a one-SD change in the polygenic risk for eveningness. The standard errors around these estimates are clustered around the inferred family ID (see Supplementary Appendix A.1) and bootstrapped with 1000 replications. To infer percentage change in sleep, estimates were also presented where the natural log of sleep duration is used as the dependent variable.

## Results

### Baseline sleep effects of night work

We start by assessing the baseline effects of night shift work on sleep duration. Table 2 shows the linear effects of the regularity of night work on sleep duration (in minutes) across nested models; for full results, including covariates, see Supplementary Appendix F. All categories of night work were associated with a reduction in sleep relative to those individuals who never/rarely work nights. Individuals who *sometimes* worked nights experienced around a 7-minute unconditional sleep penalty per night (95% confidence interval [CI] = −9:16, −5:29; *p* < 0.001), or a 4-minute penalty when all relevant covariates were added (CI = −7:24, −1:26; *p* < 0.001). Individuals who *usually* worked nights experienced a nonsignificant 4-minute unconditional sleep penalty (CI = −8:32, 1:06; *p* > 0.01), which reduced to 2 min when all covariates were added (CI = −6:51, 3:34; *p* > 0.01).

**Table 2.** Linear effect of night shift work on sleep duration (minutes:seconds)

	1		2		3		4	
	No covariates		Demographics, employment, eveningness PGS and 5 PCs (Principle Components) adjusted for		Alcohol, smoking, and snoring adjusted for		Commuting and neuroticism adjusted for	
Variable	*B*	% Change	*B*	% Change	*B*	% Change	*B*	% Change
Regularity of night work (never/rarely ref.)								
Sometimes works nights	−7:22*** [−9:55, −4:49]	−1.97%	−4:46*** [−7:17, −2:14]	−1.26%	−4:43*** [−7:15, −2:11]	−1.25%	−4:25*** [−7:24, −1:26]	−1.18%
Usually works nights	−3:43 [−8:32, 1:06]	−1.23%	−1:47 [−6:34, 2:59]	−0.65%	−1:41 [−6:28, 3:05]	−0.62%	−1:38 [−6:51, 3:34]	−0.62%
Always works nights	−15:23*** [−19:08, −11:37]	−4.25%	−12:43*** [−16:28, −8:58]	−3.46%	−12:37*** [−16:22, −8:53]	−3.43%	−12:49*** [−17:01, −8:36]	−3.47%
* N*	53 211		53 211		53 211		53 211	
* R* ^*2*^	0.003		0.027		0.028		0.036	

Beta coefficient (*B*) shows change in self-reported sleep duration (minutes:seconds). Confidence intervals, shown in parentheses, are calculated using bootstrapped standard errors (based on 1000 replications) that are clustered around the family ID. The “% Change” column shows change in the percentage of total sleep duration; this is the *B* coefficient on the natural log of sleep duration. All estimates are produced from a one-third random sample of the UK Biobank, which includes individuals between the ages of 39 and 65 who are in paid employment of at least 10 h per week. Nested models are shown which sequentially add covariates from variables from left to right. ***p < 0.00.

**Figure 1.**
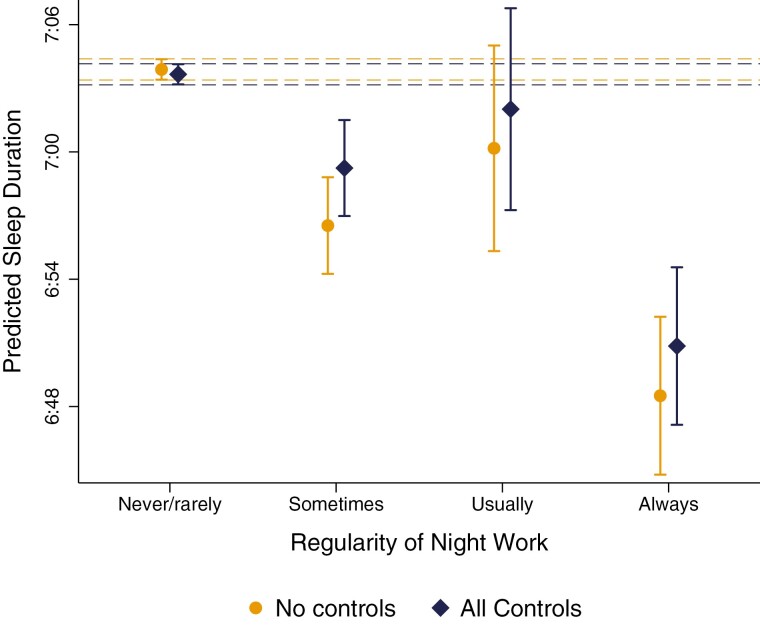
Predicted sleep duration, by regularity of night shift work. “All covariates” estimates are conditional on the full set of covariates. Dashed lines show the upper and lower confidence intervals for the reference group (never/rarely works nights); treatment groups for which confidence intervals overlap with these dashed lines show no significant difference at *a* = 0.05.

The steepest sleep penalties were by a large margin observed for those individuals who *always* worked nights, experiencing an unconditional sleep penalty of 15 min (CI = −17:58, −11:38; *p* < 0.001), or 13 min when all covariates were added (CI = −17:01, −8:36; *p* < 0.001). As shown in Table 2, this equated to a nearly 3.5% reduction in total sleep time per night. Figure 1 plots predicted sleep duration by the regularity of night work. Individuals who never/rarely worked nights get on average 7 h and 4 min of sleep per night (CI = 7:03:10, 7:04:10), while individuals who always worked nights get on average 6 h and 51 min per night (CI = 6:47:08, 6:54:34). This equated to a predicted sleep penalty of around one-and-half-hours over the week (CI = −1:59:07, −1:00:12; *p* < 0.05).

**Figure 2.**
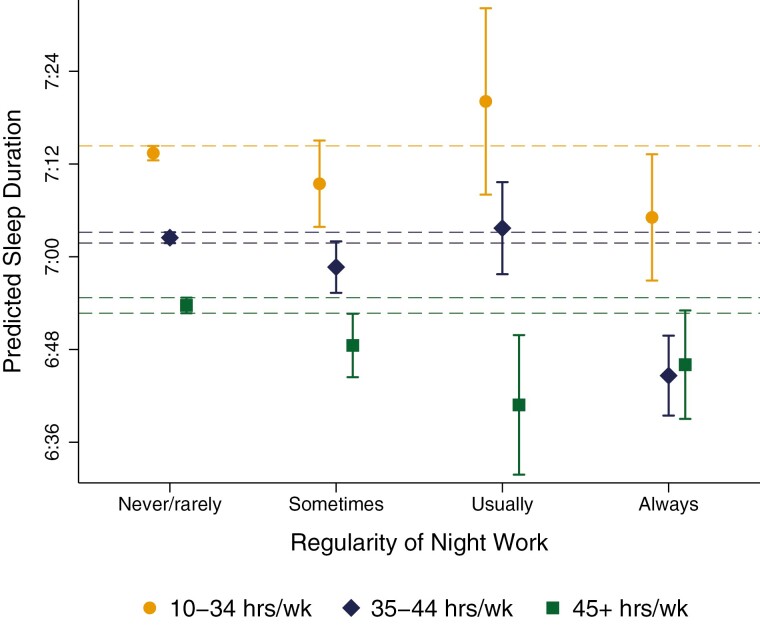
Predicted sleep duration, by regularity of night shift work and work hours. All estimates are conditional on the full set of covariates. Vertical capped lines show 95% confidence intervals, which are calculated using bootstrapped standard errors based on 1000 replications that are clustered around the family ID. Dashed lines show the upper and lower confidence intervals for the reference group (never/rarely works nights); treatment groups for which confidence intervals overlap with these dashed lines show no significant difference at *a* = 0.05.

Figure 2 plots predicted sleep duration over the regularity of night shift work stratifying by weekly working hours. For individuals working part-time (i.e. < 35 h/wk), night work was associated with no significant sleep penalties relative to those who *never/rarely* worked these hours. Among individuals working full-time (i.e. 35–44 h/wk), a significant sleep penalty of roughly 18 min was observed for those who *always* worked nights (CI = −23:29, −11:50; *p* < 0.001). In contrast, among individuals working long hours (≥45 hours/week), only *usual* night work was associated with a significant sleep penalty (*B* = −12:56; CI = −23:00, −2:53; *p* < 0.05), while the sleep penalty for *always* night work did not reach significance (*B* = −7:28; CI = −15:34, 0:39; *p* > 0.1). This latter trend, while partially tied to a lack of power, may reflect a positive selection bias into long-hours night shift arrangements. That is, the individuals who *always* work nights in excess of 45 h per week may be selective of those best suited to cope in these arrangements, with the others having selected out [44].

**Figure 3.**
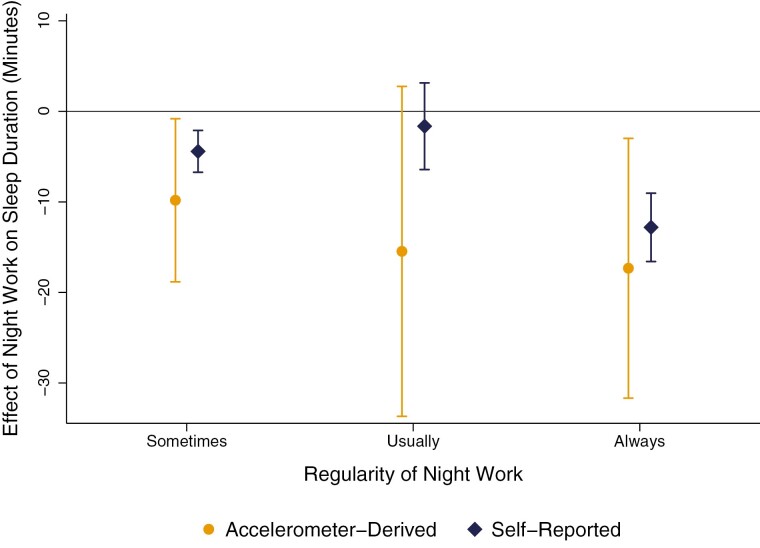
Comparing marginal effects of night shift work on self-reported and accelerometer-derived sleep duration. All estimates are conditional on the full set of covariates. Vertical capped lines show 95% confidence intervals, which are calculated using bootstrapped standard errors based on 1000 replications that are clustered around the family ID.

Sensitivity analyses showed consistent patterns when an actigraphy-derived sleep duration measure was used (see Figure 3). However, actigraphy-derived measures tended to produce higher estimates of the sleep penalty associated with night work, although confidence intervals overlapped with self-reported sleep duration estimates. Supplementary Table S10 in Appendix G shows that the largest sleep penalties were again observed for individuals *always* working nights, who experienced an actigraphy-derived sleep penalty of 17 minutes per night (CI = −32:03, −2:36; *p* < 0.05), or a penalty of roughly two hours over the week (CI = −3:44:21, −0:18:12). *Sometimes* night work was associated with a 10-minute sleep penalty per night (CI = −18:59, −0:40; *p* < 0.05), and the sleep penalty for *usual* night work was again not significant (*B* = −15:28, CI = −33:23, 2:27; *p* > 0.1). Since night shift work is more prevalent among males than females, we also performed a sensitivity analyses by sex (Supplementary Appendix H). We found that while men on average slept less, the sleep penalties associated with night work did not significantly vary by sex (Supplementary Table S11 and Appendix H). We also compared sleep penalties of night work in male dominated jobs to those penalties in female-dominated jobs within our sample (Supplementary Table S12 and Figures S6 and S7, Appendix H). While sleep duration was lower in male-dominated jobs, there were no systematic differences in the sleep penalties associated with night shift work between job groups.

### Modifying effect of eveningness

We then assessed whether and how these sleep penalties might vary depending on an individual’s genetic propensity for eveningness. Table 3 shows the effects of night shift work interacted with the genetic propensity for eveningness (i.e. the standardized eveningness PGS) across nested models. A first observation was that the eveningness PGS appeared to have no direct effect on sleep duration, suggesting that eveningness does not necessarily lead to reductions in sleep. Crucially, we can also see that the PGS eveningness had a significant buffering effect on the night shift work sleep penalty, but only for those individuals who *always* worked nights.

**Table 3.** Linear effect of night shift work interacted with PGS for eveningness on sleep duration (minutes:seconds)

	1		2		3		4	
	5 PCs adjusted for		Demographics and employment adjusted for		Alcohol, smoking, and snoring adjusted for		Commuting and neuroticism adjusted for	
Variable	*B*	% Change	*B*	% Change	*B*	% Change	*B*	% Change
Regularity of night work (never/rarely ref.)								
Sometimes works nights	−7:20*** [−9:53, −4:47]	−1.96%	−4:45*** [−7:16, −2:14]	−1.26%	−4:43*** [−7:14, −2:11]	−1.25%	−4:25*** [−6:55, −1:53]	−1.18%
Usually works nights	−3:48 [−8:37, 1:02]	−1.24%	−1:50 [−6:37, 2:57]	−0.66%	−1:43 [−6:30, 3:03]	−0.63%	1:41 [−6:25, 3:03]	−0.63%
Always works nights	−15:29*** [−19:14, −11:44]	−4.27%	−12:46*** [−16:31, −9:01]	−3.48%	−12:41*** [−16:25, −8:56]	−3.44%	−13:07*** [−17:15, −9:00]	−3.49%
Eveningness PGS	−0:04 [−0:34, 0:25]	−0.01%	−0:02 [−0:32, 0:27]	0.00%	−0:01 [−0:31, 0:28]	0.00%	0:00 [−0:29, 0:29]	0.01%
Eveningness PGS * night work (never/rarely ref.)								
Sometimes works nights	1:01 [−1:21, 3:22]	0.29%	0:44 [−1:34, 3:03]	0.22%	0:42 [−1:37, 3:01]	0.20%	0:42 [−1:37, 3:01]	0.20%
Usually works nights	−1:38 [−6:04, 2:47]	−0.43%	−1:27 [−5:46, 2:52]	−0.38%	−1:33 [−5:52, 2:46]	−0.40%	−1:22 [−5:38, 2:55]	−0.35%
Always works nights	4:17* [0:51, 7:44]	1.09%	3:43* [0:16, 7:09]	0.96%	3:43* [0:17, 7:08]	0.95%	3:37* [0:10, 7:04]	0.93%
* N*	53 211		53 211		53 211		53 211	
* R* ^*2*^	0.003		0.028		0.028		0.036	

Beta coefficient (*B*) shows change in self-reported sleep duration (minutes:seconds). Confidence intervals, shown in parentheses, are calculated using bootstrapped standard errors (based on 1000 replications) that are clustered around the family ID. The “% Change” column shows change in the percentage of total sleep duration; this is the *B* coefficient on the natural log of sleep duration. All estimates are produced from a one-third random sample of the UK Biobank, which includes individuals between the ages of 39 and 65 who are in paid employment of at least 10 h per week. Nested models are shown which sequentially add covariates from variables from left to right. *p < 0.05, **p < 0.01, ***p < 0.001.

In column 4, we see that on average (i.e. when the standardized eveningness PGS = 0) individuals who *always* worked nights experienced around a 13-minute sleep penalty relative to those who *never/rarely* worked nights (CI = −17:15, −9:00; *p* < 0.001). However, this sleep penalty reduced by nearly 4 minutes with each one SD increase in the PGS for eveningness (CI = 0:10, 7:04; *p* < 0.05), a buffering effect which remained significant across all nested models (columns 1–4). A 1- SD increase in eveningness equated to a nearly 1% increase in total sleep for individuals who *always* worked nights and reduced the sleep penalty associated with these schedules by roughly 28%. In contrast, we observed no significant buffering effect of eveningness for those who only *sometimes* or *usually* worked nights.

**Figure 4.**
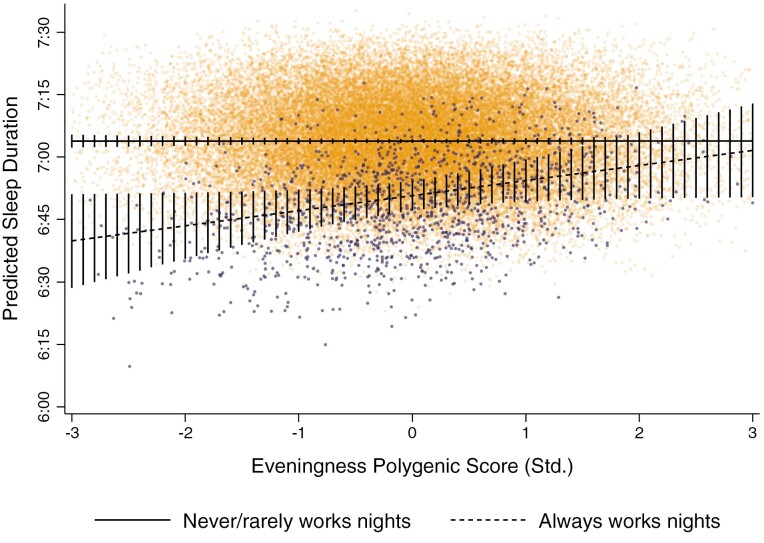
Predicted sleep duration of individuals never/rarely and always working nights, over eveningness PGS (std.). Estimates are conditional on the full list of covariates provided in Table 2. Vertical lines show 95% confidence intervals, which are calculated using bootstrapped standard errors based on 1000 replications that are clustered around the family ID.

To further depict the protective effects of eveningness, Figure 4 plotted fitted sleep duration (conditioning on all covariates) for individuals who *never/rarely* and *always* worked nights over the PGS for eveningness (for plots of *sometimes* and *usual* night work, see Supplementary Appendix I). Individuals who *always* worked nights with the lowest PGS for eveningness (3 SD’s below the mean; i.e. *extreme morning-typed individuals*) had a predicted sleep duration of around 6 h and 40 min per night (CI = 6:28:30, 6:51:09), while morning-typed counterparts who *never/rarely* worked nights slept around 7 h and 4 min night (CI = 7:02:19, 7:05:18). This equated to a 24-minute sleep penalty over the day (CI = −36:04, −11:11; *p* < 0.01), or a nearly 3-hour sleep penalty over the week (CI = −4:12:28, −1:18:17). In contrast, individuals who always worked nights with the highest PGS for eveningness (3 SD’s above the mean; i.e. *extreme evening-typed individuals*) slept around 7 h and 2 min per night and experienced no significant sleep penalty relative to similarly evening-typed counterparts who *never/rarely* worked nights (*B* = −2:16; CI = −14:56, 10:25; *p* > 0.1). Notably, Figure 4 also shows that no significant sleep penalties were observed for individuals who *always* worked nights with a PGS for eveningness at least 1.6 SD’s above the mean.

**Figure 5.**
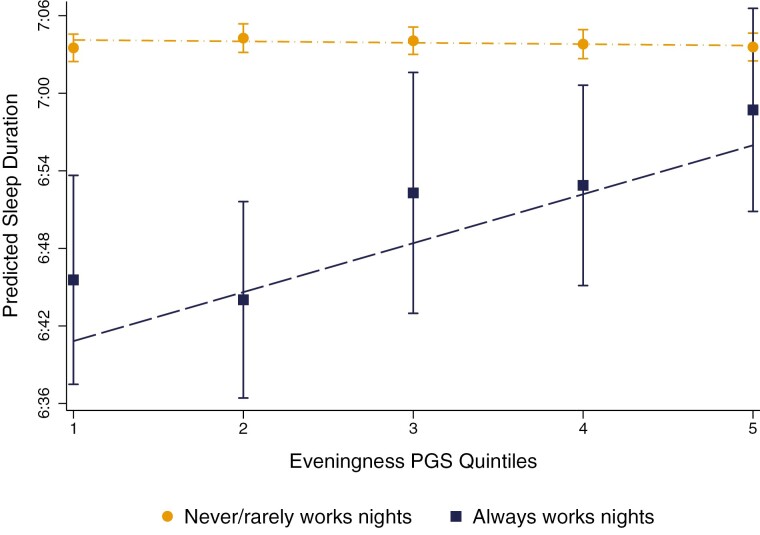
Predicted sleep duration of individuals never/rarely and always working nights, over 5 eveningness PGS quintiles. Estimates are conditional on the full list of covariates provided in Table 2. Vertical lines show 95% confidence intervals, which are calculated using bootstrapped standard errors based on 1000 replications that are clustered around the family ID.

Sensitivity analyses where quadratic and cubic specifications of the eveningness PGS were interacted with night work (see Supplementary Appendix J) show no improvement in model fit, suggesting that the moderating effect of eveningness was relatively linear. Similarly, fitting sleep duration over quintiles of the eveningness PGS (see Figure 5) showed a robust and linear patterning of effects. Individuals ranking in the lowest 20% of the PGS for eveningness who *always* worked nights slept around 6 hours and 35 minutes per night (CI = 6:37:04, 6:54:04) and experienced an 18-minute sleep penalty relative to counterparts who *never/rarely* worked nights (CI = −27:30, −8:24; *p* < 0.01). In contrast, individuals ranking in the highest 20% of the PGS for eveningness had a predicted sleep duration of 6 h and 59 min and experienced no significant sleep penalty (*B* = −4:52; CI = −13:41, 3:56; *p* > 0.1).

**Figure 6.**
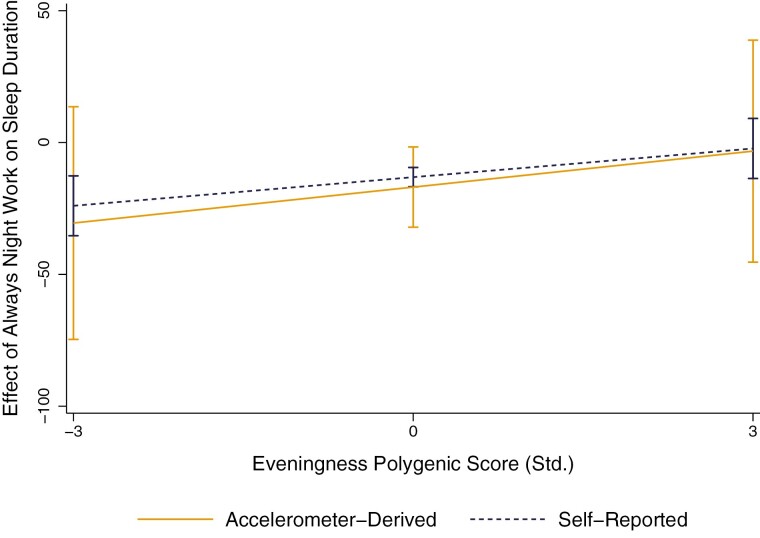
Marginal effects of always night work on self-reported and actigraphy-derived sleep duration, over eveningness PGS. Estimates are conditional on the full list of covariates provided in Table 2. Vertical lines show 95% confidence intervals, which are calculated using bootstrapped standard errors based on 1000 replications that are clustered around the family ID. Marginal effects represent the change in minutes of sleep relative to those who never/rarely work nights.

Comparing the buffering effects of eveningness when an actigraphy-derived measure of sleep duration was used (Supplementary Appendix K) revealed a highly similar patterning of effects. Figure 6 shows the buffering effect of the eveningness PGS on the *always* night work sleep penalty, illustrating a remarkably similar trend when an actigraphy-derived sleep duration measure was used. However, the interaction coefficients between the eveningness PGS and *always* night work no longer reached significance, likely due to a loss of power and the small number of regular night workers who wore accelerometers.

Our additional analysis by sex did not reveal any sex-specific patterns in the protective effects including always night shift workers (Supplementary Table S12, Appendix H). The magnitude of the interaction term is the same size was in the main analyses, but not significant, likely due to reduced sample size and power.

### Stratifying by work hours

Table 4 shows the effects of night work interacted with the PGS for eveningness on sleep duration over categories of working hours (10–34 h/wk; 35–44 h/wk; ≥45 h/wk). The eveningness PGS only had a buffering effect on *always* night work for those individuals working more than 45 h per week. In column 6, we can see that on average (i.e. when the standardized eveningness PGS = 0), individuals who *always* worked nights more than 45 h per week experienced around an 7-minute sleep penalty relative to those who never/rarely worked nights (CI = −12:26, −1:14; *p* < 0.05). However, this sleep penalty reduced by roughly 11 minutes with each one-SD increase in the PGS for eveningness (CI = 5:50, 16:44; *p* < 0.001). This equated to a nearly 165% sleep-penalty reduction with each one-SD increase in the eveningness PGS.

**Table 4.** Linear effect of night shift work interacted with PGS for eveningness on sleep duration, over work hours

Variable	10–34 h/wk		35–44 h/wk		≥45 h/wk	
	1	2	3	4	5	6
	No covariates	All covariates	No covariates	All covariates	No covariates	All covariates
	*B*	*B*	*B*	*B*	*B*	*B*
Regularity of night work (never/rarely ref.)						
Sometimes works nights	−2:28 [−7:50, 2:55]	−3:46 [−9:08, 1:37]	−4:29** [−7:41, −1:16]	−3:46* [−7:01, −0:32]	−6:35*** [−10:19, −2:52]	−5:01** [−8:48, −1:14]
Usually works nights	7:38 [−2:24, 17:41]	7:15 [−2:44, 17:15]	0:07 [−5:23, 5:35]	1:01 [−4:29, 6:30]	−14:16*** [−22:12, −6:20]	−13:02** [−21:01, −5:04]
Always works nights	−8:35* [−15:40, −1:29]	−8:47* [−15:53, −1:41]	−19:03*** [−23:22, −14:44]	−17:38*** [−21:59, −13:17]	−8:03** [−13:30, −2:36]	−6:50* [−12:26, −1:14]
Eveningness PGS	−0:14 [−1:11, 0:41]	−0:08 [−1:04, 0:47]	0:20 [−0:22, 1:02]	0:25 [−0:17, 1:07]	−0:40 [−1:41, 0:22]	−0:40 [−1:41, 0:22]
Eveningness PGS * night work (never/rarely ref.)						
Sometimes works nights	−3:39 [−8:55, 1:36]	−3:31 [−8:43, 1:41]	1:23 [−1:49, 4:35]	1:05 [−2:05, 4:15]	2:41 [−1:04, 6:27]	2:41 [−1:02, 6:25]
Usually works nights	6:17 [−4:10, 16:44]	7:17 [−3:04, 17:39]	−4:35 [−10:10, 0:58]	−4:58+ [−10:29, 0:33]	1:25 [−6:05, 8:55]	1:52 [−5:35, 9:18]
Always works nights	4:33 [−2:36, 11:42]	4:34 [−2:31, 11:39]	−0:44 [−4:59, 3:30]	−1:01 [−5:13, 3:12]	11:45*** [6:15, 17:14]	11:17*** [5:50, 16:44]
* N*	15 675	15 675	25 177	25 177	12 359	12 359
* R* ^*2*^	0.001	0.022	0.004	0.021	0.005	0.025

Beta coefficient (*B*) shows change in self-reported sleep duration (minutes:seconds). Confidence intervals, shown in parentheses, are calculated using bootstrapped standard errors (based on 1000 replications) that are clustered around the family ID. All estimates are produced from a one-third random sample of the UK Biobank, which includes individuals between the ages of 39 and 65 who are in paid employment of at least 10 h per week. Nested models are shown which sequentially add covariates from variables from left to right. +*p* < 0.01, *p < 0.05, **p < 0.01, ***p < 0.000.

**Figure 7.**
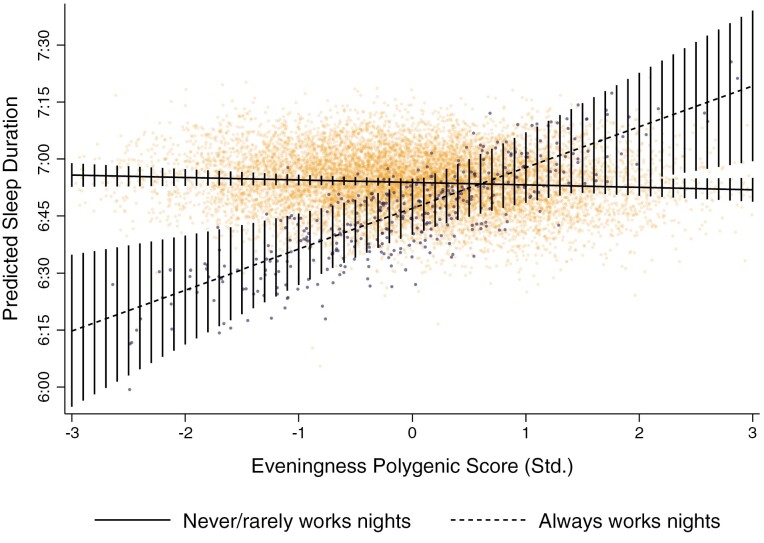
**Predicted** sleep duration of individuals never/rarely and always working nights (≥45 h/wk), over PGS for eveningness. Estimates are conditional on the full set of covariates. Vertical lines depict 95% confidence intervals constructed from bootstrapped standard errors (1000 replications) clustered around the family ID.

Figure 7 plots the predicted sleep durations of individuals working in excess of 45 h per week who either *never/rarely* or *always* worked nights over the PGS for eveningness. Extreme morning-typed individuals (i.e. those with an eveningness PGS 3 SD’s below the mean) who always worked nights more than 45 h per week slept on average 6 h and 15 min per night (CI = 5:55:18, 6:35:06), while morning-typed counterparts who *never/rarely* work nights slept around 6 hours and 56 minutes per night (CI = 6:52:40, 6:58:56). This equates to a nearly 41-minute sleep penalty per night (CI = −1:03:38, −17:34; *p* < 0.001) or to a nearly 5-hour sleep penalty over the week (CI = −7:25:26, −2:02:34).

However, as can be seen in Figure 7, individuals at or above the mean in eveningness (i.e. standardized eveningness PGS ≥ 0) experienced no significant sleep penalty at *a* = 0.05, and individuals with a PGS at least 2 SDs above the mean in fact experienced a significant sleep reward relative to counterparts who *never/rarely* worked nights. For example, extreme evening-typed individuals (i.e. those with an eveningness PGS 3 SD’s above the mean) who always worked nights more than 45 h per week slept on average 7 h and 19 min per night (CI = 6:28:25, 7:39:23), while similarly evening-typed counterparts who *never/rarely* worked nights slept around 6 h and 52 min per night (CI = 6:48:43, 6:54:56). This equated to a sleep reward of around 27 min per night (CI = 3:29, 50:40; *p* < 0.05) or more than 3 h over the week (CI = 24:23, 5:54:40). Sensitivity analyses showed a similar pattern when an actigraphy-derived measure of sleep duration was used (see Supplementary Appendix K, Table S14).

## Discussion

Consistent with past research, we found that night work was associated with significant sleep penalties, the largest of which were observed for individuals that always work nights, experiencing a sleep penalty of 13 min per night (or 1-and-a-half hours per week) relative to those who never worked such hours. Our results are consistent with previous studies, which found that shift workers reported a 15-minute shorter sleep duration compared to daytime workers and have an increased risk insomnia and falling asleep at work [45]. This is given the fact that sleep plays an essential role for physical and mental health [46, 47]. Sleep-related problems have been shown to have a negative impact on cognitive functioning, accident rates and interpersonal conflict [48]. However, these estimates were attenuated to the greatest extent once employment conditions were considered, namely: working hours per week and, to a lesser but still substantial extent, distance of weekly commutes. There, demographic characteristics play a role, particularly the age group of a regular shift worker. Although night shift work was more common among males, we did not find any significant differences in sleep penalties among males and females. Also, no differences were observed sleep penalties between male- and female-dominated jobs.

We also found that a genetic propensity for eveningness had a protective effect on night workers. A one-SD increase in the PGS for eveningness was associated with a 28% reduction in the *always* night work sleep penalty per night. This protective effect of eveningness was strongest for those working the longest night shifts but did not vary between males and females: individuals who always worked nights ≥45 hours per week with the lowest PGS for eveningness experiencing a sleep penalty of around 41 min per night, while counterparts with the highest PGS for eveningness in fact experienced a sleep reward of around 27 min per night.

As more and more firms continue to rely on shift work to expand hours of operation, it is essential to develop a better understanding of how to design these arrangements and to source workers such that negative health side-effects are minimized. As Phillips [49] notes, working in a night schedule does not necessarily lead to adverse health consequences. Similarly, being a night owl should in itself not be a problem. Negative health effects may instead come in part from having to live and work in arrangements that are incongruent with one’s own clock. Findings from this study suggest that sleep duration is not as impacted for night workers who have an evening or owl clock. Future research could examine the mechanisms behind this, such as whether and how workers adjust, quality of life or other health outcomes. Existing mixed-method and longitudinal research has also found that there is a selection effect of who remains in these jobs due their adverse effects on health, personal and family life [44, 50]. While there is no panacea, such trends indicate that solutions to the negative side-effects of night shift work should take individual differences in chronotype into account.

There are several limitations of our study. First, the UKB does not include longer term information on whether individuals are in rotating or irregular shifts and includes the information on the primary job only. We, therefore, do not capture multiple and part time jobs undertaken by those who might “sometimes” work at night. Such a division could be important to explain the differences that we found between those working “sometimes” and “usually” and associated sleep penalties. We note that those who work multiple jobs make up only around 3.6% of the UK workforce, which has remained relatively stable over time [51]. We also note that working part-time in one’s main job is not associated with probabilities to be involved in shift work [52]. Hence, this data limitation is unlikely to substantially bias or influence our results.

Second, due to data limitations, we do not have a repeated measurement on sleep and thus our study cannot provide implications for the longitudinal effects of shift work and genetic factors on sleep. Third, as noted previously, our sample may impact the generalizability of our findings. The UKB includes participants who are more likely to be older, female, fewer ethnic minorities and live in less socioeconomically deprived areas [53]. We also selected only those with valid PGSs of White British ancestry and due to the ages of data collection in the study, those aged 39–65. Previous studies have shown that those who work in jobs with nonstandard hours are overrepresented by ethnic minorities, who also have additional labor force disadvantages [54]. This suggests our results may be conservative estimates. Another potential limitation is that we only examined workers aged 39–65. There is evidence that older shift workers have shorter and more disturbed sleeping patterns, suggesting conversely to ethnicity, that sleep problems in our study have the potential to be slightly overestimated [55, 56]. Working conditions of those in night shifts may have also varied across time, with research showing that more protective labor market regulations and collective bargaining agreements shield workers from potential detrimental effects of these nonstandard working times [57].

Future research could focus on examining the compounded or intersectional inequality of impacts across different groups in relation to occupation, age and race, ethnicity, socioeconomic group and variation in employment protection. This field of research also needs more diversity in genetic samples, to expand beyond primarily those of European ancestry. It could explore further the pathways through which individuals are best able to align their actual and chronotypically optimal work schedules as well as the downstream consequences of working schedules. Studying such questions could shed new light on some of the hidden mechanisms underlying health disparities in the labor market and could ultimately inform human resource strategies to improve health outcomes and reduce turnover among night shift workers.

## Supplementary Material

zsad023_suppl_Supplementary_Material

## Data Availability

The data underlying this article were provided by UK Biobank under project ID 32696. Interested researchers should contact the UK Biobank for access to data. More information can be found here: https://www.ukbiobank.ac.uk/

## References

[1] Straif K , et al.; WHO International Agency For Research on Cancer Monograph Working Group. Carcinogenicity of shift-work, painting, and fire-fighting. Lancet Oncol. 2007;8(12):1065–1066. doi:10.1016/S1470-2045(07)70373-X. 19271347

[2] Office for National Statistics. *Shift Workers in the Public Sector*. *ONS Report*. https://www.ons.gov.uk/employmentandlabourmarket/peopleinwork/employmentandemployeetypes/adhocs/13069shiftworkersinthepublicsector; 2020. Accessed June 16, 2021.

[3] Foster RG , et al. Sleep and circadian rhythm disruption in social jetlag and mental illness. Prog Mol Biol Transl Sci. 2013;119:325–346. 23899602 10.1016/B978-0-12-396971-2.00011-7

[4] Gu F , et al. Total and cause-specific mortality of U.S. nurses working rotating night shifts. Am J Prev Med. 2015;48(3):241–252. doi:10.1016/j.amepre.2014.10.018. 25576495 PMC4339532

[5] Lee HY , et al. Association between shift work and severity of depressive symptoms among female nurses: the Korea Nurses’ Health Study. J Nurs Manag. 2016;24(2):192–200. doi:10.1111/jonm.12298. 25950801

[6] Vetter C , et al. Night shift work, genetic risk, and type 2 diabetes in the UK biobank. Diabetes Care. 2018;41(4):762–769. doi:10.2337/dc17-1933. 29440150 PMC5860836

[7] Hansen J. Night shift work and risk of breast cancer. Curr Environ Heal Reports. 2017;4(3):325–339. doi:10.1007/s40572-017-0155-y. 28770538

[8] Papantoniou K , et al. Night shift work, chronotype and prostate cancer risk in the MCC-Spain case-control study. Int J Cancer. 2015;137(5):1147–1157. doi:10.1002/ijc.29400. 25530021

[9] Yuan X , et al. Night shift work increases the risks of multiple primary cancers in women: a systematic review and meta-analysis of 61 articles. Cancer Epidemiol Biomarkers Prev. 2018;27(1):25–40. doi:10.1158/1055-9965.EPI-17-0221. 29311165

[10] Rajaratnam SMW , et al. Sleep loss and circadian disruption in shift work: health burden and management. Med J Aust. 2013;199(S8):S11–S15. doi:10.5694/mja13.10561. 24138359

[11] Boudreau P , et al. Circadian adaptation to night shift work influences sleep, performance, mood and the autonomic modulation of the heart. PLoS One. 2013;8(7):e70813. doi:10.1371/journal.pone.0070813. 23923024 PMC3724779

[12] Wittmann M , et al. Social jetlag: misalignment of biological and social time. Chronobiol Int. 2006;23(1–2):497–509. doi:10.1080/07420520500545979. 16687322

[13] Haus EL , et al. Shift work and cancer risk: potential mechanistic roles of circadian disruption, light at night, and sleep deprivation. Sleep Med Rev. 2013;17(4):273–284. doi:10.1016/j.smrv.2012.08.003. 23137527

[14] Kervezee L , et al. Simulated night shift work induces circadian misalignment of the human peripheral blood mononuclear cell transcriptome. Proc Natl Acad Sci. 2018;115(21):5540–5545. doi:10.1073/pnas.1720719115. 29735673 PMC6003514

[15] Schwartz J , et al. Shift work sleep disorder. Drugs. 2006;66(18):2357–2370. doi:10.2165/00003495-200666180-00007. 17181377

[16] Boivin DB , et al. Impacts of shift work on sleep and circadian rhythms. Pathol Biol. 2014;62(5):292–301. doi:10.1016/j.patbio.2014.08.001. 25246026

[17] Kervezee L , et al. Metabolic and cardiovascular consequences of shift work: the role of circadian disruption and sleep disturbances. Eur J Neurosci. 2020;51(1):396–412. doi:10.1111/ejn.14216. 30357975

[18] Chellappa SL , et al. Circadian misalignment increases mood vulnerability in simulated shift work. Sci Rep. 2020;10(1):18614. doi:10.1038/s41598-020-75245-9. 33122670 PMC7596056

[19] Richter K , et al. Shiftwork and alcohol consumption: a systematic review of the literature. Eur Addict Res. 2021;27(1):9–15. doi:10.1159/000507573. 32454482

[20] Booker LA , et al. Exploring the associations between shift work disorder, depression, anxiety and sick leave taken amongst nurses. J Sleep Res. 2020;29(3). doi:10.1111/jsr.12872. 31144389

[21] Kennedy KER , et al. Long sleep: is there such thing as too much of a good thing? Curr Sleep Med Reports. 2022;8(3):35–41. doi:10.1007/s40675-022-00224-7.

[22] Roenneberg T , et al. Life between clocks: Daily temporal patterns of human chronotypes. J Biol Rhythms. 2003;18(1):80–90. doi:10.1177/0748730402239679. 12568247

[23] Achari KV , et al. Comparison of distributions of morningness–eveningness among populations of shift workers on varied work patterns in different organizations. Biol Rhythm Res. 2012;43(3):235–248. doi:10.1080/09291016.2011.571025.

[24] Adan A , et al. Circadian typology: a comprehensive review. Chronobiol Int. 2012;29(9):1153–1175. doi:10.3109/07420528.2012.719971. 23004349

[25] Gamble KL , et al. Shift work in nurses: contribution of phenotypes and genotypes to adaptation (factors that contribute to shift-work adaptation). PLoS One. 2011;6(4):e18395. doi:10.1371/journal.pone.0018395. 21533241 PMC3076422

[26] Juda M , et al. Chronotype modulates sleep duration, sleep quality, and social jet lag in shift-workers. J Biol Rhythms. 2013;28(2):141–151. doi:10.1177/0748730412475042. 23606613

[27] Saksvik IB , et al. Individual differences in tolerance to shift work—A systematic review. Sleep Med Rev. 2011;15(4):221–235. doi:10.1016/j.smrv.2010.07.002. 20851006

[28] Vetter C , et al. Aligning work and circadian time in shift workers improves sleep and reduces circadian disruption. Curr Biol. 2015;25(7):907–911. doi:10.1016/j.cub.2015.01.064. 25772446

[29] Härmä M. Individual differences in tolerance to shiftwork: a review. Ergonomics. 1993;36(1-3):101–109. doi:10.1080/00140139308967860. 8440205

[30] Roenneberg T , et al. The human circadian clock entrains to sun time. Curr Biol. 2007;17(2):R4444–R4R45. doi:10.1016/j.cub.2006.12.011. 17240323

[31] Davey Smith G , et al. Mendelian randomization: genetic anchors for causal inference in epidemiological studies. Hum Mol Genet. 2014;23(1):R8989–R8R98. doi:10.1093/hmg/ddu328. PMC4170722 25064373

[32] Mills MC , Barban N, Tropf FC. An Introduction to Statistical Genetic Data Analysis. Cambridge, MA: The MIT Press; 2020.

[33] Mills MC , et al. The GWAS diversity monitor tracks diversity by disease in real time. Nat Genet. 2020;52(3):242–243. doi:10.1038/s41588-020-0580-y. 32139905

[34] Mills MC , et al. A scientometric review of genome-wide association studies. Commun Biol. 2019;2(1). doi:10.1038/s42003-018-0261-x. PMC6323052 30623105

[35] Jones SE , et al. Genome-wide association analyses in 128,266 individuals identifies new morningness and sleep duration loci. PLoS Genet. 2016;12(8):e1006125. doi:10.1371/journal.pgen.1006125. 27494321 PMC4975467

[36] Jones SE , et al. Genome-wide association analyses of chronotype in 697,828 individuals provides insights into circadian rhythms. Nat Commun. 2019;10(1):343. doi:10.1038/s41467-018-08259-7. 30696823 PMC6351539

[37] Purcell SM , et al. PLINK: a tool set for whole-genome association and population-based linkage analyses. Am J Hum Genet. 2007;81(3):559–575. doi:10.1086/519795. 17701901 PMC1950838

[38] Chang CC , et al. Second-generation PLINK: rising to the challenge of larger and richer datasets. GigaScience. 2015;4:7. doi:10.1186/s13742-015-0047-8. 25722852 PMC4342193

[39] Euesdon J , et al. Polygenic risk score software. Bioinformatics. 2015;31(9):1466–1468. doi:10.1093/bioinformatics/btu848. 25550326 PMC4410663

[40] Bulik-Sullivan BK , et al. LD Score regression distinguishes confounding from polygenicity in genome-wide association studies. Nat Genet. 2015;47(3):291–295. 25642630 10.1038/ng.3211 PMC4495769

[41] Lane JM , et al. Genome-wide association analysis identifies novel loci for chronotype in 100,420 individuals from the UK Biobank. Nat Commun. 2016;7:10889. doi:10.1038/ncomms10889. 26955885 PMC4786869

[42] Garrido MM , et al. Methods for constructing and assessing propensity scores. Health Serv Res. 2014;49(5):1701–1720. doi:10.1111/1475-6773.12182. 24779867 PMC4213057

[43] Mosca M , et al. Sleep duration, snoring habits, and cardiovascular disease risk factors in an ethnically diverse population. J Cardiovasc Nurs. 2012;27(3):263–269. doi:10.1097/JCN.0b013e31821e7ad1. 21743341 PMC3627372

[44] Mills M , et al. Nonstandard work schedules and partnership quality: quantitative and qualitative findings. J Marriage Fam. 2010;72(4):860–875. doi:10.1111/j.1741-3737.2010.00735.x.

[45] Kecklund G , et al. Health consequences of shift work and insufficient sleep. BMJ. 2016;355:i5210. doi:10.1136/bmj.i5210. 27803010

[46] Purta R , et al. Experiences measuring sleep and physical activity patterns across a large college cohort with fitbits. In: Proceedings of the 2016 ACM International Symposium on Wearable Computers. New York, NY: ACM; 2016:28–35. doi:10.1145/2971763.2971767

[47] Schwartz J , et al. Neurophysiology of sleep and wakefulness: basic science and clinical implications. Curr Neuropharmacol. 2008;6(4):367–378. doi:10.2174/157015908787386050. 19587857 PMC2701283

[48] Hafner M , Stepanek M, Taylor J, Troxel WM, van Stolk C. Why sleep matters-the economic costs of insufficient sleep: a cross-country comparative analysis. Rand Heal Q. 2017;6(4):11. 10.7249/rhq-06-04-11 PMC5627640 28983434

[49] Phillips ML. Circadian rhythms: of owls, larks and alarm clocks. Nature. 2009;458(7235):142–144. doi:10.1038/458142a. 19279605

[50] Täht K , et al. Nonstandard work schedules, couple desynchronization, and parent–child interaction. J Fam Issues. 2012;33(8):1054–1087. doi:10.1177/0192513X11424260.

[51] Corlett A , Finch D. *Double Take: Workers With Multiple Jobs and Reforms to National Insurance* . London, UK: Resolution Foundation; 2016. https://www.resolutionfoundation.org/app/uploads/2016/11/Double-take.pdf.

[52] Bosworth D. Shiftwork in the UK: evidence from the LFS. Appl Econ. 1994;26(6):617–626. doi:10.1080/00036849400000032.

[53] Fry A , et al. Comparison of sociodemographic and health-related characteristics of UK biobank participants with those of the general population. Am J Epidemiol. 2017;186(9):1026–1034. doi:10.1093/aje/kwx246. 28641372 PMC5860371

[54] Presser HB. Race-ethnic and gender differences in nonstandard work shifts. Work Occup. 2003;30(4):412–439. doi:10.1177/0730888403256055.

[55] Blok MM , et al. What is the evidence for less shift work tolerance in older workers? Ergonomics. 2011;54(3):221–232. doi:10.1080/00140139.2010.548876. 21390952

[56] Folkard S. Shift work, safety, and aging. Chronobiol Int. 2008;25(2-3):183–198. doi:10.1080/07420520802106694. 18484360

[57] Taiji R , et al. Non-standard schedules, work-family conflict, and the moderating role of national labour context: evidence from 32 European countries. Eur Sociol Rev. 2020;36(2):179–197. doi:10.1093/esr/jcz048.

