# Supplementary material for: Gene-x-environment analysis supports protective effects of eveningness chronotype on self-reported and actigraphy-derived sleep duration among those who always work night shifts in the UK Biobank
Source: Sleep. 2023 Feb 6;46(5):zsad023. doi: 10.1093/sleep/zsad023 (PMC10171639; doi:10.1093/sleep/zsad023)
Supplement: zsad023_suppl_Supplementary_Material [file zsad023_suppl_supplementary_material.docx]

**Supplementary Material**

**Gene-X-Environment Analysis Supports Protective Effects of Eveningness Chronotype on Self-Reported and Actigraphy-derived Sleep Duration among Regular Night Shift Workers in the UK Biobank**

Evelina T. Akimova (0000-0001-8733-3745),^1,*^ Riley Taiji (0000-0002-1475-4490),^1^ Xuejie Ding (0000-0002-0280-8463),^1^ and Melinda C. Mills (0000-0003-1704-0001)^1,2,3*^

^1^Leverhulme Centre for Demographic Science & Nuffield College, University of Oxford, UK

^2^Department of Economics, Econometrics and Finance, University of Groningen, The Netherlands

^3^Department of Genetics, University Medical Centre Groningen, The Netherlands

^*^Corresponding authors:

[evelina.akimova@demography.ox.ac.uk](mailto:evelina.akimova@demography.ox.ac.uk) and [melinda.mills@demography.ox.ac.uk](mailto:melinda.mills@demography.ox.ac.uk)

42-43 Park End Street, Oxford, United Kingdom OX1 1JD

Table of Contents

[Appendix A: Sample Design Overview 5](#_Toc124536161)

[A.1. Handling of Genetically Related Individuals 5](#_Toc124536162)

[A.2. Overview of Sample Restrictions 6](#_Toc124536163)

[A.3. Overview of Sample Representativeness 8](#_Toc124536164)

[Appendix B: Overview of GWAS analysis and polygenic score calculation 11](#_Toc124536165)

[B.1 Genome-wide association analysis 11](#_Toc124536166)

[B.2 Post-GWAS analyses 12](#_Toc124536167)

[B.3 Polygenic score calculation and prediction 15](#_Toc124536168)

[Appendix C: Overview of Actigraphy-Derived Sleep Duration Measure 17](#_Toc124536169)

[Appendix D: Descriptive Statistics of Covariates 19](#_Toc124536170)

[Appendix E: Common Occupations by Night Work Category and Sleep Penalties 21](#_Toc124536171)

[Appendix F: Baseline Model Full Results 28](#_Toc124536172)

[Appendix G: Baseline Sensitivity Analyses Using Actigraphy-Derived Sleep Duration Measure 31](#_Toc124536173)

[Appendix H: Sensitivity analyses by sex 32](#_Toc124536174)

[Appendix I: Moderating Effect of Eveningness PGS on Sometimes and Usual Night Work 37](#_Toc124536175)

[Appendix J: Moderating Effect of Eveningness PGS on Always Night Work with Quadratic and Cubic Polynomials 37](#_Toc124536176)

[Appendix K: Eveningness PGS Sensitivity Analyses Using Actigraphy-Derived Sleep Duration Measure 38](#_Toc124536177)

[References 40](#_Toc124536178)

**Supplementary Tables**

Table S1. *Overview of Sample Restrictions*

Table S2. *Overview of Sample Representativeness*

Table S3. *Summary of LD score intercept, SE and mean*$\chi^{2}$

Table S4. *Comparison of Self-Reported and Accelerometer-Derived Sleep Duration*

Table S5. *Proportions/Means of Covariates, by Regularity of Night Work*

Table S6: Top 10 Common Occupations for *Sometimes* Night Work

Table S7: Top 10 Common Occupations for *Usually* Night Work

Table S8: Top 10 Common Occupations for *Always* Night Work

Table S9. *Baseline OLS Regression of Sleep Duration*

Table S10. *OLS Regression of Actigraphy-Derived Sleep Duration on Night Shift Work*

Table S11. *Most Common Occupations Involving Night Shift Work, by Sex*

Table S12. *Linear Effect of Night Shift Work Interacted with PGS for Eveningness on Sleep Duration, over Sex*

Table S13. *Linear Effect of Night Shift Work Interacted with PGS for Eveningness on Actigraphy-Derived Sleep Duration*

Table S14. *Linear Effect of Night Shift Work Interacted with PGS for Eveningness on Actigraphy-Derived Sleep Duration, over Work Hours*

**Supplementary Figures**

Figure S1*. SNP-heritability of chronotype outcomes*

Figure S2. *Genetic correlations of chronotype outcomes*

Figure S3: Effect of *Sometimes* Night Work on Sleep Duration, over 2-digit ISCO

Figure S4: Effect of *Usually* Night Work on Sleep Duration, over 2-digit ISCO

Figure S5: Effect of *Always* Night Work on Sleep Duration, over 2-digit ISCO

Figure S6. *Effect of Night Work on Sleep Duration, by Sex*

Figure S7. *Effect of Night Work on Sleep Duration in Male- and Female-Dominated Occupations*

Figure S8. *Effects of Male’s Night Work on Sleep Duration, by Occupation*

Figure S9. *Effects of Female’s Night Work on Sleep Duration, by Occupation*

Figure S10. *Predicted Sleep Duration of Individuals Never/Rarely and Sometimes Working Nights, over PGS for Eveningness (Std.)*

Figure S11. *Predicted Sleep Duration of Individuals Never/Rarely and Usually Working Nights, over PGS for Eveningness (Std.)*

Figure S12. *Predicted Sleep Duration of Individuals Never/Rarely and Always Working Nights, over PGS for Eveningness (Std.) Squared*

Figure S13. *Predicted Sleep Duration of Individuals Never/Rarely and Always Working Nights, over PGS for Eveningness (Std.) Cubed*

# Appendix A: Sample Design Overview

### A.1. Handling of Genetically Related Individuals

Including related individuals in analyses can inflate estimates of the effects of polygenic scores (PGSs).^1^ Here we discuss how we account for this. To calculate genetic relatedness, we use the KING kinship coefficient.^2^ In line with standard benchmarks, we use a coefficient threshold of >= 0.0884 to designate dyads as sufficiently related. This threshold includes 2^nd^ degree relations (e.g., uncles, aunts, grandparents, cousins) as well as closer 1^st^ degree relations (e.g., siblings, parents, twins). Of the total 502,599 individuals sampled in the UK Biobank, 67,460 individuals had a kinship coefficient >= 0.0884 with another respondent. Of these individuals, 64,614 were of White British ancestry and had heterozygosity within 3 SDs of the mean.

We placed all of these 64,614 related individuals into the prediction set and randomly sampled a further 87,252 unrelated individuals to add to this sample (see Appendix A.2). This left a non-overlapping sample of 303,672 unrelated individuals of White British ancestry and with heterozygosity within 3 SDs of the mean in the reference set to perform the GWAS on. In the prediction set, we then created a family ID unique to every grouping of individuals where relatedness was at or above our threshold of 0.0884. We identified 30,404 unique families within the prediction set, where the number of individuals nested within families ranged from 2 to 10. We then assigned a unique family ID to the remaining 87,252 unrelated individuals. To account for relatedness in the prediction set, we cluster standard errors around this family ID in all analyses.

### A.2. Overview of Sample Restrictions

The UK Biobank (UKB) provides genetic, demographic, and employment information on roughly 502,599 British respondents between the ages of 39-70.^3^ The sample restrictions imposed on this set are numbered in Table S1 and discussed in what follows. In restriction 1, we keep only individuals who had available genetic data present (*n* = 488,335). In restriction 2, we keep individuals who identify as ‘British’, ‘Irish’, or ‘Any other white background’ (*n* = 459,707); this is done to avoid spurious genetic associations induced by population stratification. In restriction 3, we restrict analysis to individuals who had levels of heterozygosity within +/- 3 SD’s from the mean (*n* = 455,538). The application of PGSs requires a non-overlapping sample upon which to perform a Genome-Wide Association Study (GWAS). In restriction 4, we therefore selected 33.3% (*n* = 151,866) of the sample for use as the prediction set and reserved the remaining 66.7% (*n* = 303,672) as a reference set to run the GWAS on. Note that all related individuals remaining after restrictions 1-3 (*n* = 64,614) were placed in the prediction set; see Appendix A.1 for details. We then randomly sampled a further 87,252 individuals to add to this prediction set such that the prediction set comprised 33.3% of 455,538 (the total sample after restrictions 1-3). The remaining 303,672 individuals were then placed in the reference set.

The analytic sample for this analysis includes individuals who, at the time of data collection, were between the ages of 39-65 and in regular paid or self-employment of at least 10 hours per week. After performing restrictions 5-7, this amounted to a sample of 79,902. We use listwise deletion on all covariates of interest. The sample loss tied to this are shown in restrictions 8-19. This led to a total analytic sample of 53,211 individuals. As can be seen, the largest sample loss occured after restrictions 18-19 when removing cases with missing values on commuting distance and the neuroticism score. In both cases, the high sample loss was tied to values marked ‘missing’ or ‘do not know’ in the source dataset. In the case of neuroticism, the measure is an index of 12 binary items in the UK Biobank questionnaire. Thus, any individuals who had a ‘missing’ or ‘do not know’ value on any of the 12 items had a missing value on the total score. In the subsequent section, we explore whether list-wise deletion on these variables affects sample representativeness.

Table S1. *Overview of Sample Restrictions*

| *#* | *Restriction* | *N* | *N Loss* | *% Loss* |
| --- | --- | --- | --- | --- |
| 0 | Total Sample (No Restrictions) | 502599 | -- | -- |
| 1 | Genetic Data Present | 488335 | -14264 | -2.84% |
| 2 | White British Ethnicity | 459707 | -28628 | -5.86% |
| 3 | Heterozygosity within 3 SDs of mean | 455538 | -4169 | -0.91% |
| 4 | Randomly select one third of sample (stratified on age, sex and night work) | 151866 | -303672 | -66.66% |
| 5 | Between ages 39-65 | 127785 | -24081 | -15.86% |
| 6 | In paid or self-employment | 83577 | -44208 | -34.60% |
| 7 | Weekly work hours > 9 and non-missing | 79902 | -3675 | -4.40% |
| 8 | Non-missing on sleep duration | 79736 | -166 | -0.21% |
| 9 | Non-missing on night work | 79710 | -26 | -0.03% |
| 10 | Non-missing on age, sex, partner present and child present | 79710 | 0 | 0.00% |
| 11 | Non-missing on years of education | 79401 | -309 | -0.39% |
| 12 | Non-missing on urban/rural | 78462 | -939 | -1.18% |
| 13 | Non-missing on occupation (SIOPS) | 74181 | -4281 | -5.46% |
| 14 | Non-missing on manual work and sedentary work | 74148 | -33 | -0.04% |
| 15 | Non-missing on tobacco use | 74125 | -23 | -0.03% |
| 16 | Non-missing on snoring | 69823 | -4302 | -5.80% |
| 17 | Non-missing on alcohol use | 69807 | -16 | -0.02% |
| 18 | Non-missing on commuting distance | 62922 | -6885 | -9.86% |
| 19 | Non-missing on neuroticism | 53211 | -9711 | -15.43% |

*Notes:* Estimates are produced from a one-third random sample of the UK Biobank.

### A.3. Overview of Sample Representativeness

In our one-third sample of the UK Biobank (UKB) participants of White British ancestry with genetic data present and heterozygosity within +/- 3 SDs of the mean (*n* = 151,866; restrictions 1-4 in Table S1), 79,902 individuals were between the ages of 39-65 and in paid or self-employment of at least 10 hours per week. This is shown under sample restrictions 5-7 in Table S1. In what follows, we will refer to this as the *Baseline Sample*.

Subsequent sample restrictions tied to list-wise deletion (restrictions 8-19 in Table S1) resulted in the loss of 26,691 individuals. To determine whether the resultant analytic sample of 53,211 individuals was representative of the greater population of interest, we compared the distributions of a host of demographic and occupational covariates between the analytic sample (shown under restriction 19 in Table S1) to the baseline sample discussed above. Results are presented in Table S2, with the B column indicating the difference between the two samples.

Our analytic sample does reasonably well in maintaining the distribution of covariates of interest seen in the baseline sample. The analytic sample appears largely representative of the larger baseline sample in terms of sleep duration and the prevalence of night work. The exceptions are that the analytic sample appears to be slightly younger (*B* = -0.19 years, *p* < 0.001), and overrepresents the percentage of individuals with partners present in the household (*B*  = 3.62%, *p* < 0.001) and the percentage of individuals with children present (*B* = 1.84%, *p* < 0.001). The analytic sample also appears to overrepresent those with higher occupational class or SIOPS values (*B* = 0.72, *p* < 0.001). As a consequence, the analytic sample also underrepresents the percentage of individuals in jobs involving manual labor (*B* = -1.00%, *p* < 0.001) and sedentary work (*B* = -1.26%, *p* < 0.001).

These differences are, however, small and the overall distributions of covariates suggest that the analytic sample does not differ substantively from the baseline sample that would be observed absent sample restrictions 7-19 in Table S1. In short, the analytic sample appears to be largely representative of the larger population of working individuals in the UK Biobank, with any minor differences reflecting systematic non-response issues endemic to large surveys in general.

Table S2. *Overview of Sample Representativeness*

|  |  | Baseline Sample | |  | Analytic Sample | |  |  |  |
| --- | --- | --- | --- | --- | --- | --- | --- | --- | --- |
| Variable | | *Mean* | *N* |  | *Mean* | *N* |  | *B* | |
| Age | | 52.01 | 79902 |  | 51.83 | 53211 |  | -0.19 | *** |
|  |  | [51.97, 52.06] |  |  | [51.77, 51.89] |  |  |  |  |
| Male | | 47.40% | 79902 |  | 47.86% | 53211 |  | 0.46% |  |
|  |  | [47.05, 47.75] |  |  | [47.44, 48.29] |  |  |  |  |
| Partner Present | | 73.86% | 79902 |  | 77.49% | 53211 |  | 3.62% | *** |
|  |  | [73.56, 74.17] |  |  | [77.13, 77.84] |  |  |  |  |
| Child Present | | 49.01% | 79902 |  | 50.85% | 53211 |  | 1.84% | *** |
|  |  | [48.66, 49.35] |  |  | [50.42, 51.27] |  |  |  |  |
| Years of Education | | 14.50 | 79578 |  | 14.58 | 53211 |  | 0.09 | * |
|  |  | [14.46, 14.53] |  |  | [14.54, 14.63] |  |  |  |  |
| Urban | | 85.28% | 78954 |  | 85.13% | 53211 |  | -0.15% |  |
|  |  | [85.03, 85.53] |  |  | [84.83, 85.43] |  |  |  |  |
| Eve. PGS (Std.) | | -0.00 | 79902 |  | -0.01 | 53211 |  | -0.01 |  |
|  |  | [-0.01, 0.01] |  |  | [-0.02, -0.00] |  |  |  |  |
| Sleep Duration (Self-Reported) | | 7:02:59 | 79736 |  | 7:03:07 | 53211 |  | 0:00:08 |  |
|  |  | [7:02:35, 7:03:23] |  |  | [7:02:38, 7:03:36] |  |  |  |  |
| Sleep Duration (Actigraphy) | | 6:32:07 | 17450 |  | 6:30:57 | 12072 |  | -0:01:10 |  |
|  |  | [6:30:57, 6:33:18] |  |  | [6:29:33, 6:32:21] |  |  |  |  |
| Neuroticism | | 4.12 | 66641 |  | 4.10 | 53211 |  | -0.02 |  |
|  |  | [4.10, 4.15] |  |  | [4.08, 4.13] |  |  |  |  |
| Work Hours | | 36.42 | 79902 |  | 36.62 | 53211 |  | 0.20 | * |
|  |  | [36.34, 36.50] |  |  | [36.52, 36.71] |  |  |  |  |
| Occupation (SIOPS) | | 47.56 | 75530 |  | 48.28 | 53211 |  | 0.72 | *** |
|  |  | [47.46, 47.66] |  |  | [48.16, 48.40] |  |  |  |  |
| Regularity of Night Work | |  |  |  |  |  |  |  |  |
|  | Never/rarely | 91.36% | 79875 |  | 91.50% | 53211 |  | 0.14% |  |
|  |  | [91.16, 91.55] |  |  | [91.26, 91.74] |  |  |  |  |
|  | Sometimes | 4.86% | 79875 |  | 4.74% | 53211 |  | -0.11% |  |
|  |  | [4.71, 5.01] |  |  | [4.56, 4.92] |  |  |  |  |
|  | Usually | 1.35% | 79875 |  | 1.32% | 53211 |  | -0.03% |  |
|  |  | [1.27, 1.43] |  |  | [1.22, 1.42] |  |  |  |  |
|  | Always | 2.43% | 79875 |  | 2.43% | 53211 |  | 0.00% |  |
|  |  | [2.33, 2.54] |  |  | [2.30, 2.56] |  |  |  |  |
| Manual Work | | 13.81% | 79875 |  | 12.82% | 53211 |  | -1.00% | *** |
|  |  | [13.57, 14.05] |  |  | [12.53, 13.10] |  |  |  |  |
| Sedentary Work | | 33.67% | 79881 |  | 32.41% | 53211 |  | -1.26% | *** |
|  |  | [33.34, 34.00] |  |  | [32.01, 32.81] |  |  |  |  |
| Weekly Commute Distance (miles) | | 48.19 | 71899 |  | 48.64 | 53211 |  | 0.45 |  |
|  |  | [47.46, 48.92] |  |  | [47.84, 49.44] |  |  |  |  |
| Current Smoker | | 7.83% | 79873 |  | 7.44% | 53211 |  | -0.38% | + |
|  |  | [7.64, 8.01] |  |  | [7.22, 7.67] |  |  |  |  |
| Snores | | 38.32% | 75222 |  | 38.31% | 53211 |  | -0.01% |  |
|  |  | [37.98, 38.67] |  |  | [37.90, 38.73] |  |  |  |  |
| Alcohol Consumption | |  |  |  |  |  |  |  |  |
|  | Never | 4.73% | 79876 |  | 4.52% | 53211 |  | -0.21% |  |
|  |  | [4.58, 4.87] |  |  | [4.35, 4.70] |  |  |  |  |
|  | Special occasions | 9.21% | 79876 |  | 8.76% | 53211 |  | -0.45% | * |
|  |  | [9.01, 9.41] |  |  | [8.52, 9.00] |  |  |  |  |
|  | One to three times a month | 12.21% | 79876 |  | 12.17% | 53211 |  | -0.04% |  |
|  |  | [11.98, 12.43] |  |  | [11.89, 12.44] |  |  |  |  |
|  | Once or twice a week | 28.87% | 79876 |  | 29.09% | 53211 |  | 0.22% |  |
|  |  | [28.55, 29.18] |  |  | [28.70, 29.47] |  |  |  |  |
|  | Three or four times a week | 25.62% | 79876 |  | 26.13% | 53211 |  | 0.51% |  |
|  |  | [25.32, 25.92] |  |  | [25.76, 26.51] |  |  |  |  |
|  | Daily or almost daily | 19.37% | 79876 |  | 19.33% | 53211 |  | -0.04% |  |
|  |  | [19.10, 19.64] |  |  | [19.00, 19.67] |  |  |  |  |

Baseline sample represents UK Biobank sample after sample restrictions 1-7 in Table S1. Analytic sample reflects UK Biobank sample after restrictions 1-19 in Table S2.

# Appendix B: Overview of GWAS analysis and polygenic score calculation

### B.1 Genome-wide association analysis

The use of PGSs requires a non-overlapping sample with associated genome-wide association study.^1^ Since Jones et al. (2016, 2019)^4,5^ GWASs include UK Biobank participants, we performed our own primary GWAS on eveningness. In doing so, we randomly selected 33.3% (n = 151,866) of the sample for use as the prediction set and reserved the remaining 66.7% (n = 303,672) as the reference set to run the GWAS.

In terms of phenotypic variable definition, we analysed a single relevant measure of chronotype available in UK Biobank which is data field no. 1180. We closely followed the approach used in Jones et al. (2016, 2019).^4,5^ Accordingly, self-reported eveningness was derived from a survey item asking respondents to report whether they, “…consider [themselves] to be”: (1) *definitely a morning person*, (2) *more a morning than evening person*, (3) *more an evening than a morning person*, (4) *definitely an evening person*, or (5) *do not know*. We coded these responses -2, -1, 1, 2 and 0, respectively, which is a reverse code of Jones et al. (2016, 2019).^4,5^ We, therefore, expect our PGS to be negatively associated with the ones based on Jones et al. (2016, 2019)^4,5^ GWASs.

Our calculations based on linear model association tests with covariates as implemented in PLINK software,^6,7^ with association testing based on v3 imputed data. PLINK software is available at [www.cog-genomics.org/plink/1.9/](http://www.cog-genomics.org/plink/1.9/). Thus, we modelled eveningness as a linear outcome and adjusted for age, sex, the first 5 genetic principal components, assessment centre (categorical), and a derived variable representing the genotyping release (categorical; UKBiLEVE array, UKB Axiom array interim release, and UKB Axiom array full release) on the sample of European ancestry individuals.

We also applied a range of quality controls. We included SNPs with missing rate less than or equal to 0.05 per SNP and less than or equal to 0.03 per individual. HWE filter was set to $\leq$ 0.00001 and MAF $\geq$0.01. In total, it resulted in 5,666,911 autosomal SNPs. We excluded individuals with a genetic relatedness greater than or equal to 2^nd^ degree (KING kinship coefficient $\geq$ 0.0884) and individuals with heterozygosity greater than 3 s.d. from the mean.

### B.2 Post-GWAS analyses

We used LDSC software^8^ to estimate LD-score regression for eveningness phenotype, calculate SNP-heritabilities for all chronotype phenotypes. The primary eveningness based on two-thirds of UK Biobank sample GWAS, and two morningness phenotypes based on UK Biobank summary statistics provided in Jones et al. (2016)^4^ and newer version of the GWAS with a larger sample size provided in Jones et al. (2019).^5^ We also use LD-score regression to compare our GWAS to Jones et al (2016, 2019)^4,5^ by quantifying genetic overlap between them. LDSC software is available at https://github.com/bulik/ldsc. For these analyses, LD scores were computed with genotypes from the European-ancestry samples in the 1000 Genomes Project using only HapMap3 SNPs (MAF > 0.01). We used the “eur_w_ld_chr” files of LD Scores provided in Finucane et al.^9^ They are available at <https://data.broadinstitute.org/alkesgroup/LDSCORE/eur_w_ld_chr.tar.bz2>.

Supplementary Table S3 below demonstrates the results of LD-score regression analysis. LD score intercept is statistically different from 1 but not substantially. Our $\chi^{2}$ statistics, which is greater than 1, and ratio estimates indicate that around 6.8% of the estimated inflation of mean $\chi^{2}$ statistics is likely capturing confounding bias rather than a polygenic signal.

Table S3. *Summary of LD score intercept, SE and mean*$\chi^{2}$

|  | Phenotype: *Eveningness* |
| --- | --- |
| LD score intercept (s.e.) ^*^ | 1.0468 (0.0124) |
| Lambda GC | 1.4709 |
| Mean $\boldsymbol{\chi}^{\boldsymbol{2}}$ | 1.6006 |
| Ratio (s.e.) ^*^ | 0.0683 (0.0158) |

*Note: 95% CI standard errors.*

SNP-heritability for all three chronotype phenotypes are presented in Supplementary Figure S1. All measures exhibit SNP-heritability significantly larger than zero, and computed estimates for Jones et al.^4,5^ GWASs fell in the 95% CI ranges reported by the authors and not statistically different from those. As expected, the SNP-heritability estimate based on our GWAS of eveningness is smaller than of those from Jones et al.^4,5^ since we have the smallest sample size across compared GWASs. Also, heritability of morningness from the second GWAS from Jones et al. (2019)^5^ is the largest, which is in line with our expectations given that it is the most recent and largest GWAS of chronotype to date. We take into consideration these findings and apply the thresholding method for polygenic score construction to maximise the predictive power of our score. Another important note is that our results are the most sensitive towards statistical power to detect small signals, and we thus restricted it to conduct a wider range of explanatory analyses.

Figure S1. *SNP-heritability of chronotype outcomes*

Perhaps the most sensible comparison for our case is genetic correlation estimates. While we have less predictive power (but not critically less), we want to ensure that the directions of our estimates are aligned with the existing GWASs. Genetic correlation is a valuable tool since the polygenic score is a quantitative metric. The genetic overlap of chronotype phenotypes is reported in Supplementary Figure S2. Here we observe genetic correlations of -1 between our GWAS and Jones et al.^4,5^ which makes our estimates analytically identical to those from previous GWASs. Negative correlation is expected due to reverse coding. Overall, it provides assurance that our polygenic score is a valuable tool for our analytic purposes. Additionally, we provide the summary statistics of our GWAS, when one can find our site estimates. Here, an important caveat is that it cannot be directly used for UK Biobank predictions by others, since we split the sample and cannot release the list of random individual IDs used in the discovery stage.

Figure S2. *Genetic correlations of chronotype outcomes*

### B.3 Polygenic score calculation and prediction

The main method of constructing the polygenic score was pruning and thresholding using PRSice software.^10^ PRSice software is available at <https://choishingwan.github.io/PRSice/> (2.3.0 version). The PGS was calculated using a prespecified threshold of p = 0.8 and with the software default values for clumping (a radius of 250 kb and r^2^=0.1). The *p*-value was specified to maximise a predictive power of the score (a best-fit-inferred p-value threshold was 0.08). Overall, a total of 340,381 variants were included after clumping. Weights were based on GWAS analysis results performed on randomly selected two thirds of UK Biobank sample described earlier.

The final PGS performed reasonably well in predicting self-reported eveningness, with an R-squared of 0.021 (2%) and an F-statistic of 3290.31 (*p* < 0.001). The final score was standardized to a cross-sample mean of 0.

# Appendix C: Overview of Actigraphy-Derived Sleep Duration Measure

The UK Biobank provided wrist-worn accelerometers to 103,695 individuals. For more details, see <https://www.ukbiobank.ac.uk/activity-monitor/> and related publications.^11^ 30,530 of these individuals were contained in our prediction set. We further restricted this group to those that were genotyped, in paid employment of at least 10 hours per weekd, had non-missing values on covariates of interest and wore the accelerometers for at least 3 of the seven days (*n* = 12,072).

On this subset, we derived a measure of sleep using acceleration averages (measured in milligravities) for each of the 24 hours of the day. For example, as we restrict analysis to individuals who wore their accelerometers for at least 3 of the 7 days, the acceleration average for hour 00:00 to 00:59 (i.e., 12:00am to 1:00am) would capture the average movement for this hour across all days that the accelerometer was worn. We set cases where the hourly acceleration average was equal to zero as missing as it was assumed that the accelerometer was not worn for this hour.

To infer a sleeping state for a given hour, we use a threshold calculated as the average of the 95^th^ percentiles of acceleration between the hours of 03:00-04:00. This is based on the assumption that roughly 95% of our sample will be asleep during these hours. Indeed, just over 96% of our sample works night shift either “Never/rarely” or “Sometimes”. We coded each of the 24 hours for which a resting state was inferred as one, and code the remaining hours as zero. We then summed these 24 dichotomous resting state variables to create a measure of average hourly sleep duration. As with the self-reported sleep duration measure, we capped accelerometer-inferred sleep duration at between 3-12 hours/day to minimize the effect of arbitrary variation in the upper and lower bounds.

Table S4, reports the means of self-reported and accelerometer-derived sleep duration as well as the correlation between the two. Mean sleep duration inferred from accelerometers (*M* = 6:46:08; CI = 6:45:14, 6:47:03) is around 25 minutes less than that inferred from self-reports (*M* = 7:10:01; CI = 7:09:41, 7:10:21). The two measures are also significantly but relatively weakly correlated (*r* = 0.19, *p* < 0.001). Sensitivity analyses adjusting the acceleration threshold to infer sleeping states do not improve this correlation nor do they reduce the gap in mean sleeping duration.

While this is an imperfect measure of sleeping duration, we argue it should provide a rough benchmark of objective resting duration against which to test the general accuracy of self-reported sleep duration. Whilst previous studies have used machine learning to infer more accurate measures of sleeping duration from the UK Biobank,^12^ we unfortunately did not have access to the untruncated raw acceleration data nor did we have access to the camera footage to infer a ground truth. Thus, we argue that this measure should be sufficient for our limited needs as a robustness measure.

Table S4. *Comparison of Self-Reported and Accelerometer-Derived Sleep Duration*

|  | Sleep Duration (hours:minutes:seconds) | |  |
| --- | --- | --- | --- |
| Measure | *Mean* | *95% CI* | *N* |
| Self-Reported | 7:10:01 | [7:09:41, 7:10:21] | 53,211 |
| Accelerometer-Inferred | 6:46:08 | [6:45:14, 6:47:03] | 12,072 |
| Correlation | 0.19 | *** |  |

*Notes:* Estimates are produced from a one-third random sample of the UK Biobank. The sample is further restricted to individuals between the ages of 39-65 who are in paid employment of at least 10 hours per week. + p < 0.1, * p < 0.05, ** p < 0.01, *** p < 0.001.

# Appendix D: Descriptive Statistics of Covariates

Table S5. *Proportions/Means of Covariates, by Regularity of Night Work*

|  |  | Regularity of Night Shift Work | | | | | | |
| --- | --- | --- | --- | --- | --- | --- | --- | --- |
| Variable | | Never/rarely |  | Sometimes |  | Usually |  | Always |
| Age Group | |  |  |  |  |  |  |  |
|  | 39-40 | 21.18% |  | 26.56% |  | 29.44% |  | 25.19% |
|  |  | [20.89, 21.47] |  | [25.17, 27.94] |  | [26.72, 32.17] |  | [23.26, 27.13] |
|  | 46-50 | 20.25% |  | 23.99% |  | 24.17% |  | 23.75% |
|  |  | [19.97, 20.54] |  | [22.65, 25.33] |  | [21.61, 26.72] |  | [21.86, 25.65] |
|  | 51-55 | 22.77% |  | 22.58% |  | 21.76% |  | 21.59% |
|  |  | [22.47, 23.07] |  | [21.27, 23.90] |  | [19.29, 24.22] |  | [19.75, 23.42] |
|  | 56-60 | 22.07% |  | 17.33% |  | 15.56% |  | 19.01% |
|  |  | [21.78, 22.37] |  | [16.14, 18.52] |  | [13.39, 17.72] |  | [17.26, 20.76] |
|  | 61-65 | 13.72% |  | 9.54% |  | 9.07% |  | 10.46% |
|  |  | [13.47, 13.96] |  | [8.61, 10.46] |  | [7.36, 10.79] |  | [9.10, 11.82] |
| Men | | 45.33% |  | 62.65% |  | 63.89% |  | 62.24% |
|  |  | [44.98, 45.69] |  | [61.13, 64.17] |  | [61.02, 66.76] |  | [60.08, 64.39] |
| Partner present | | 74.52% |  | 70.24% |  | 67.04% |  | 69.71% |
|  |  | [74.21, 74.83] |  | [68.80, 71.67] |  | [64.23, 69.85] |  | [67.66, 71.75] |
| Child/grandchild present | | 48.75% |  | 48.88% |  | 48.33% |  | 50.28% |
|  |  | [48.39, 49.11] |  | [47.32, 50.45] |  | [45.35, 51.32] |  | [48.06, 52.51] |
| Years of Education | | 14.65 |  | 13.45 |  | 12.92 |  | 12.39 |
|  |  | [14.61, 14.68] |  | [13.30, 13.59] |  | [12.65, 13.19] |  | [12.19, 12.59] |
| Urban | | 84.93% |  | 86.81% |  | 87.36% |  | 89.35% |
|  |  | [84.68, 85.19] |  | [85.74, 87.87] |  | [85.36, 89.36] |  | [87.97, 90.72] |
| Occupational Class (SIOPS) | | 48.30 |  | 41.81 |  | 38.38 |  | 36.75 |
|  |  | [48.19, 48.40] |  | [41.35, 42.26] |  | [37.59, 39.16] |  | [36.18, 37.31] |
| Work Hours | |  |  |  |  |  |  |  |
|  | 10-34 work hours/wk | 31.79% |  | 18.85% |  | 18.60% |  | 21.04% |
|  |  | [31.45, 32.13] |  | [17.62, 20.09] |  | [16.27, 20.93] |  | [19.22, 22.86] |
|  | 35-44 work hours/wk | 46.34% |  | 44.94% |  | 52.65% |  | 47.72% |
|  |  | [45.98, 46.70] |  | [43.38, 46.51] |  | [49.66, 55.64] |  | [45.49, 49.95] |
|  | > than 44 work hours/wk | 21.87% |  | 36.20% |  | 28.74% |  | 31.24% |
|  |  | [21.57, 22.17] |  | [34.69, 37.72] |  | [26.03, 31.45] |  | [29.17, 33.31] |
| Manual Labour Job | | 11.96% |  | 28.99% |  | 31.48% |  | 40.33% |
|  |  | [11.73, 12.19] |  | [27.57, 30.42] |  | [28.71, 34.26] |  | [38.14, 42.52] |
| Sedentary Job | | 31.60% |  | 55.83% |  | 62.93% |  | 66.03% |
|  |  | [31.27, 31.93] |  | [54.28, 57.39] |  | [60.04, 65.82] |  | [63.92, 68.14] |
| Current Smoker | | 7.28% |  | 12.41% |  | 11.48% |  | 14.34% |
|  |  | [7.09, 7.46] |  | [11.38, 13.45] |  | [9.58, 13.39] |  | [12.78, 15.90] |
| Snores | | 37.72% |  | 44.20% |  | 39.62% |  | 43.22% |
|  |  | [37.36, 38.08] |  | [42.59, 45.81] |  | [36.58, 42.66] |  | [40.92, 45.52] |
| Alcohol Consumption | |  |  |  |  |  |  |  |
|  | Never | 4.76% |  | 4.62% |  | 4.72% |  | 6.19% |
|  |  | [4.61, 4.91] |  | [3.96, 5.28] |  | [3.46, 5.99] |  | [5.12, 7.26] |
|  | Special occasions | 9.03% |  | 10.73% |  | 10.19% |  | 14.54% |
|  |  | [8.83, 9.24] |  | [9.75, 11.70] |  | [8.38, 11.99] |  | [12.97, 16.11] |
|  | One to three times a month | 12.02% |  | 13.42% |  | 12.96% |  | 15.11% |
|  |  | [11.79, 12.25] |  | [12.35, 14.49] |  | [10.96, 14.97] |  | [13.52, 16.71] |
|  | Once or twice a week | 28.62% |  | 29.87% |  | 30.28% |  | 31.25% |
|  |  | [28.30, 28.95] |  | [28.43, 31.31] |  | [27.53, 33.02] |  | [29.19, 33.32] |
|  | Three or four times a week | 25.69% |  | 24.25% |  | 24.63% |  | 21.40% |
|  |  | [25.38, 26.00] |  | [22.90, 25.60] |  | [22.06, 27.20] |  | [19.58, 23.23] |
|  | Daily or almost daily | 19.88% |  | 17.12% |  | 17.22% |  | 11.50% |
|  |  | [19.59, 20.16] |  | [15.93, 18.30] |  | [14.97, 19.48] |  | [10.08, 12.92] |
| Commuting Distance | |  |  |  |  |  |  |  |
|  | 0-15 miles/wk | 34.50% |  | 29.45% |  | 29.34% |  | 37.34% |
|  |  | [34.14, 34.87] |  | [27.94, 30.97] |  | [26.52, 32.15] |  | [35.13, 39.55] |
|  | 16-40 miles/wk | 33.19% |  | 32.05% |  | 34.09% |  | 32.41% |
|  |  | [32.83, 33.55] |  | [30.50, 33.61] |  | [31.16, 37.02] |  | [30.28, 34.55] |
|  | > 40 miles/wk | 32.31% |  | 38.49% |  | 36.57% |  | 30.25% |
|  |  | [31.95, 32.67] |  | [36.87, 40.11] |  | [33.59, 39.55] |  | [28.15, 32.35] |
| Neuroticism Score | | 4.12 |  | 4.15 |  | 4.16 |  | 4.29 |
|  |  | [4.09, 4.14] |  | [4.03, 4.26] |  | [3.94, 4.37] |  | [4.13, 4.45] |
| *N* | | 75390 |  | 3901 |  | 1080 |  | 1941 |

*Notes:* Estimates are produced from a one-third random sample of the UK Biobank. The sample is further restricted to individuals between the ages of 39-65 who are in paid employment of at least 10 hours per week. 95% confidence intervals are shown in parentheses.

# Appendix E: Common Occupations by Night Work Category and Sleep Penalties

Our analytic approach of modelling sleep penalties does not assume the linearity of night shift work levels, i.e., we treat regularity of night work categorically; hence, “*never/rarely”, “sometimes”, “usually”*, and “*always* works night shift” not as a continuum. In this section, we demonstrate that such a decision is based on the notion that the types of jobs in different shift work categories vary remarkably (Tables S6-S8). Moreover, not only do occupations differ, but the occupations where sleep penalties are most pronounced differ as well. Figures S3-S5 show plotted coefficients which represent the linear effect of the focal night work category on self-reported sleep duration conditional on the full set of covariates shown in the manuscript. In simpler terms, coefficients show how much less (more) sleep per night a night worker gets than an equivalent worker who never works such hours.

Table S6: Top 10 Common Occupations for *Sometimes* Night Work

| *Sometimes Works Nights* | | | | | | |
| --- | --- | --- | --- | --- | --- | --- |
| Women | | |  | Men | | |
| Occupation | N | Pct. |  | Occupation | N | Pct. |
| nursing and midwifery associate professionals | 378 | 27.9 |  | motor-vehicle drivers | 262 | 11 |
| personal care and related workers | 296 | 21.9 |  | protective services workers | 172 | 7.19 |
| protective services workers | 54 | 3.99 |  | building finishers and related trades workers | 156 | 6.52 |
| general managers | 45 | 3.32 |  | production and operations department managers | 149 | 6.23 |
| health professionals (except nursing) | 45 | 3.32 |  | messengers, porters, doorkeepers and related workers | 101 | 4.22 |
| production and operations department managers | 44 | 3.25 |  | machinery mechanics and fitters | 92 | 3.85 |
| shop salespersons and demonstrators | 40 | 2.95 |  | architects, engineers and related professionals | 87 | 3.64 |
| social work associate professionals | 32 | 2.36 |  | personal care and related workers | 75 | 3.14 |
| optical and electronic equipment operators | 28 | 2.07 |  | other department managers | 66 | 2.76 |
| other office clerks | 26 | 1.92 |  | building frame and related trades workers | 64 | 2.68 |

Table S7: Top 10 Common Occupations for *Usually* Night Work

| *Usually Works Nights* | | | | | | |
| --- | --- | --- | --- | --- | --- | --- |
| Women | | |  | Men | | |
| Occupation | N | Pct. |  | Occupation | N | Pct. |
| nursing and midwifery associate professionals | 117 | 33.2 |  | motor-vehicle drivers | 71 | 10.4 |
| personal care and related workers | 103 | 29.3 |  | messengers, porters, doorkeepers and related workers | 64 | 9.37 |
| client information clerks | 13 | 3.69 |  | protective services workers | 50 | 7.32 |
| travel attendants and related workers | 11 | 3.12 |  | machinery mechanics and fitters | 50 | 7.32 |
| general managers | 10 | 2.84 |  | personal care and related workers | 29 | 4.25 |
| protective services workers | 10 | 2.84 |  | building finishers and related trades workers | 26 | 3.81 |
| other office clerks | 9 | 2.56 |  | general managers | 24 | 3.51 |
| health professionals (except nursing) | 6 | 1.7 |  | manufacturing labourers | 24 | 3.51 |
| modern health associate professionals (except nursing) | 6 | 1.7 |  | production and operations department managers | 23 | 3.37 |
| social work associate professionals | 6 | 1.7 |  | chemical-processing-plant operators | 23 | 3.37 |

Table S8: Top 10 Common Occupations for *Always* Night Work

| *Always Works Nights* | | | | | | |
| --- | --- | --- | --- | --- | --- | --- |
| Women | | |  | Men | | |
| Occupation | N | Pct. |  | Occupation | N | Pct. |
| personal care and related workers | 217 | 31.8 |  | motor-vehicle drivers | 137 | 11 |
| nursing and midwifery associate professionals | 203 | 29.7 |  | messengers, porters, doorkeepers and related workers | 126 | 10.1 |
| shop salespersons and demonstrators | 34 | 4.98 |  | protective services workers | 105 | 8.43 |
| client information clerks | 29 | 4.25 |  | machinery mechanics and fitters | 85 | 6.83 |
| general managers | 16 | 2.34 |  | transport labourers and freight handlers | 60 | 4.82 |
| messengers, porters, doorkeepers and related workers | 16 | 2.34 |  | manufacturing labourers | 47 | 3.78 |
| protective services workers | 15 | 2.2 |  | production and operations department managers | 40 | 3.21 |
| domestic and related helpers, cleaners and launderers | 13 | 1.9 |  | personal care and related workers | 40 | 3.21 |
| production and operations department managers | 12 | 1.76 |  | architects, engineers and related professionals | 36 | 2.89 |
| social work associate professionals | 11 | 1.61 |  | building finishers and related trades workers | 31 | 2.49 |

Figure S3: Effect of *Sometimes* Night Work on Sleep Duration, over 2-digit ISCO


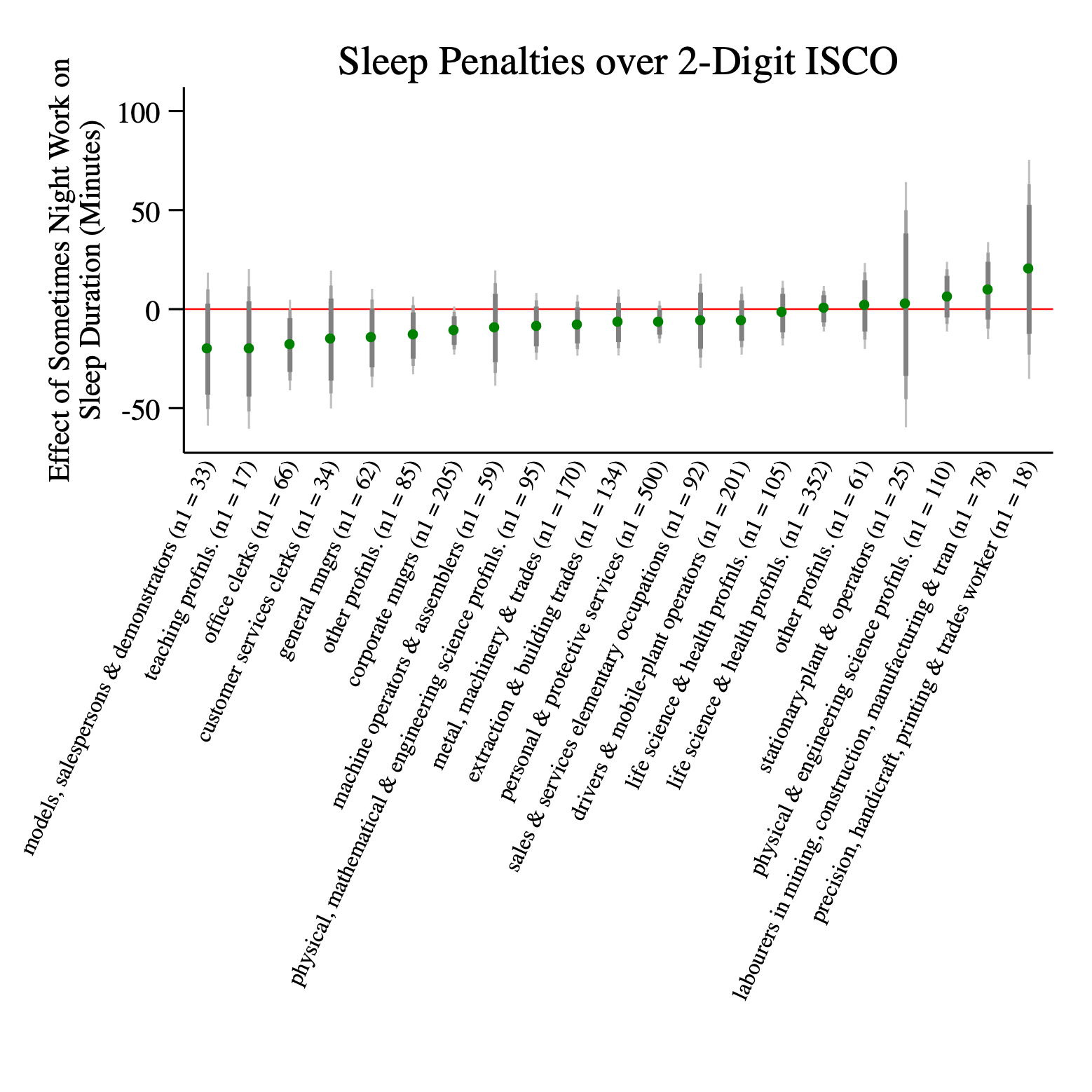


Note: ISCO refers to the International Standard Classification of Occupations.^13^

Figure S4: Effect of *Usually* Night Work on Sleep Duration, over 2-digit ISCO


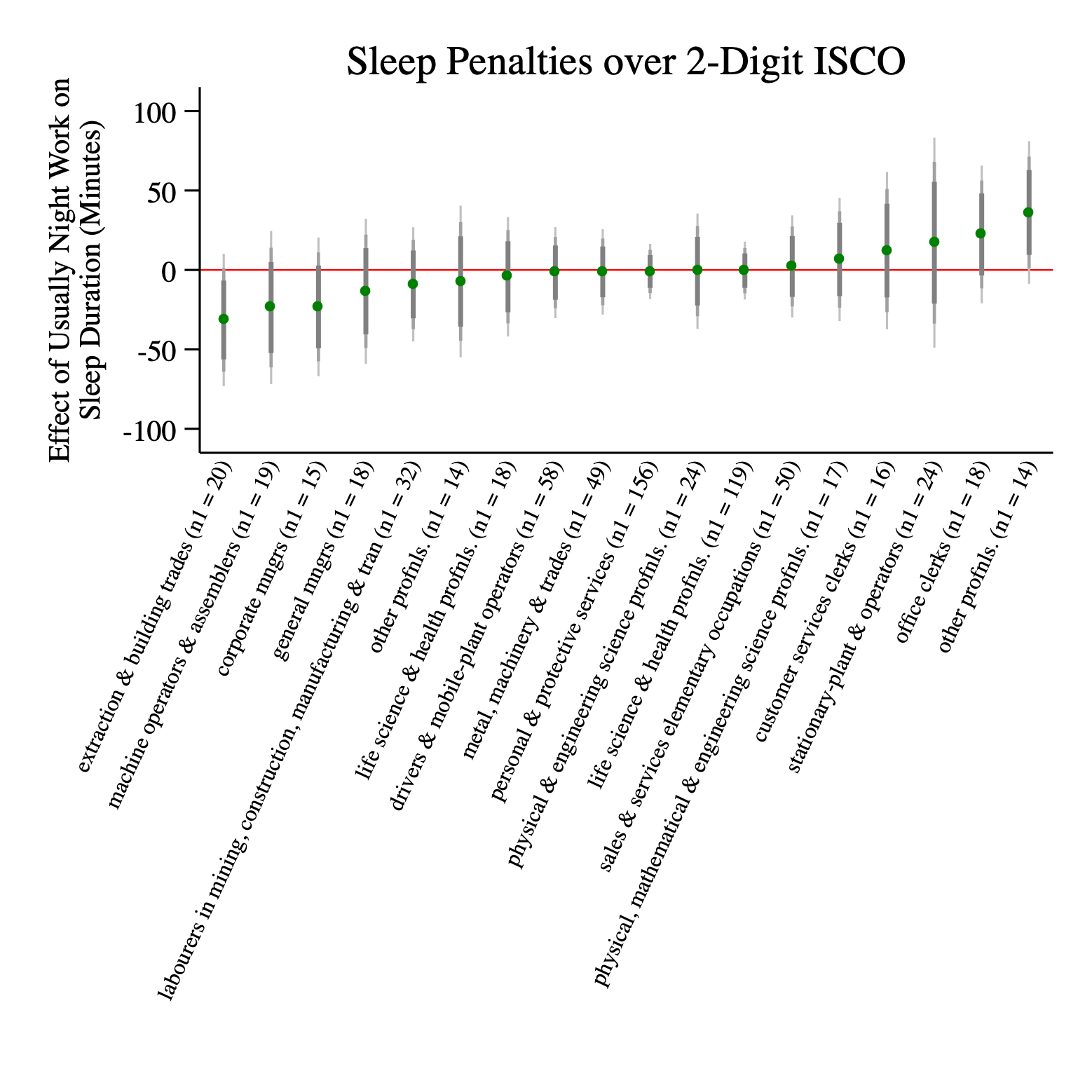


Note: ISCO refers to the International Standard Classification of Occupations.^13^

Figure S5: Effect of *Always* Night Work on Sleep Duration, over 2-digit ISCO


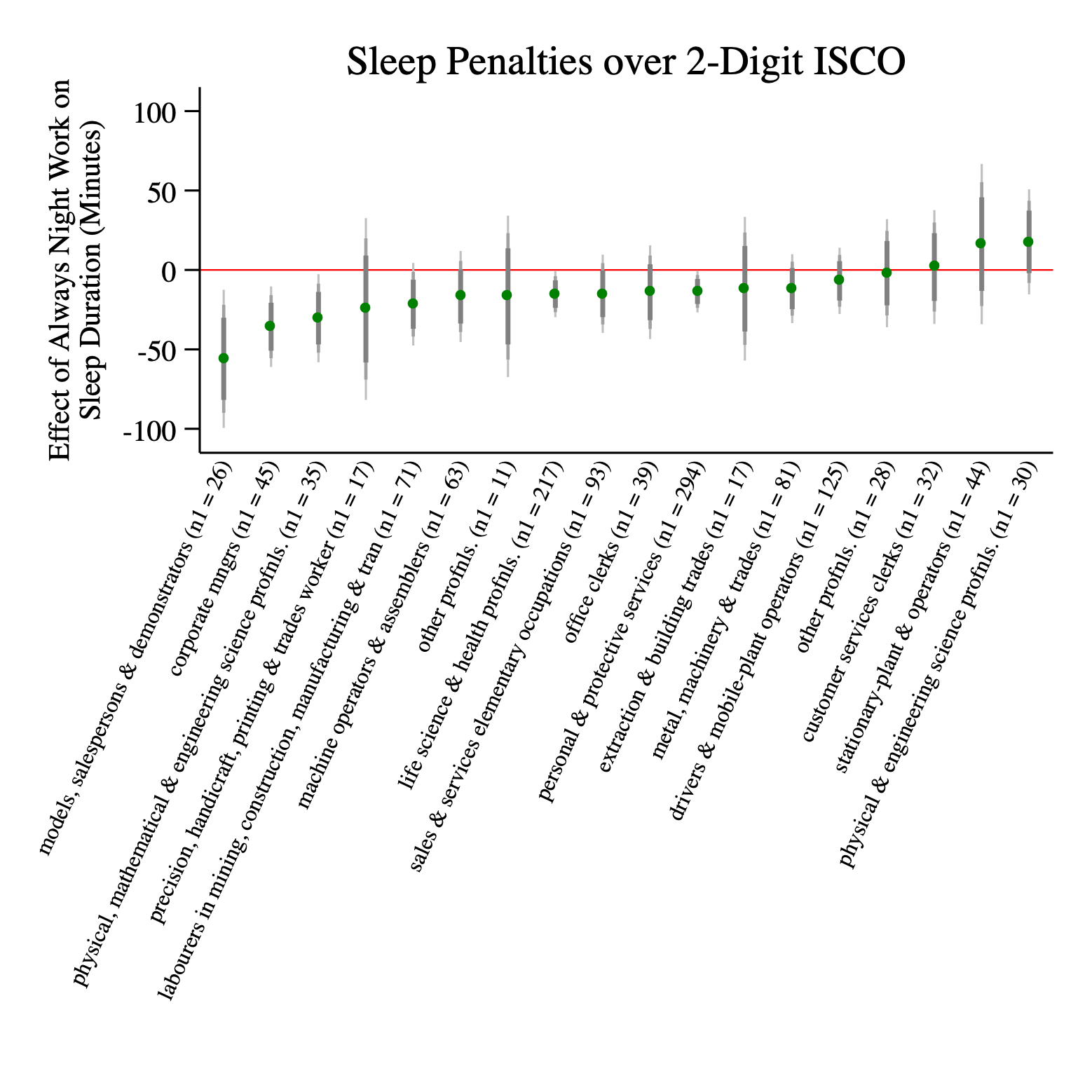


Note: ISCO refers to the International Standard Classification of Occupations.^13^

# Appendix F: Baseline Model Full Results

Table S9. *Baseline OLS Regression of Sleep Duration*

|  |  | 1 | | |  | 2 | | |  | 3 | | |  | 4 | | |
| --- | --- | --- | --- | --- | --- | --- | --- | --- | --- | --- | --- | --- | --- | --- | --- | --- |
|  |  | No Covariates | | |  | Demographics, Employment and Eve. PGS Adjusted for | | |  | Alcohol, Smoking and Snoring Adjusted for | | |  | Commuting and Neuroticism Adjusted for | | |
| Variable | | *B* | | *% Change* |  | *B* | | *% Change* |  | *B* | | *% Change* |  | *B* | | *% Change* |
| Regularity of Night Work (Never/rarely ref.) | |  |  |  |  |  |  |  |  |  |  |  |  |  |  |  |
|  | Sometimes works nights | -7:22 | *** | -1.97% |  | -4:46 | *** | -1.26% |  | -4:43 | *** | -1.25% |  | -4:25 | *** | -1.18% |
|  |  | [-9:55, -4:49] | |  |  | [-7:17, -2:14] | |  |  | [-7:15, -2:11] | |  |  | [-7:24, -1:26] | |  |
|  | Usually works nights | -3:43 |  | -1.23% |  | -1:47 |  | -0.65% |  | -1:41 |  | -0.62% |  | -1:38 |  | -0.62% |
|  |  | [-8:32, 1:06] | |  |  | [-6:34, 2:59] | |  |  | [-6:28, 3:05] | |  |  | [-6:51, 3:34] | |  |
|  | Always works nights | -15:23 | *** | -4.25% |  | -12:43 | *** | -3.46% |  | -12:37 | *** | -3.43% |  | -12:49 | *** | -3.47% |
|  |  | [-19:08, -11:37] | |  |  | [-16:28, -8:58] | |  |  | [-16:22, -8:53] | |  |  | [-17:01, -8;36] | |  |
| Eveningness PGS | |  |  |  |  | 0:04 |  | 0.03% |  | 0:05 |  | 0.03% |  | 0:07 |  | 0.04% |
|  |  |  |  |  |  | [-0:24, 0:32] | |  |  | [-0:23, 0:33] | |  |  | [-0:22, 0:34] | |  |
| PC 1 | |  |  |  |  | 0:01 |  | 0.00% |  | 0:01 |  | 0.00% |  | 0:00 |  | 0.00% |
|  |  |  |  |  |  | [-0:10, 0:10] | |  |  | [-0:10, 0:10] | |  |  | [-0:10, 0:10] | |  |
| PC 2 | |  |  |  |  | 0:02 |  | 0.01% |  | 0:02 |  | 0.01% |  | 0:02 |  | 0.01% |
|  |  |  |  |  |  | [-0:10, 0:13] | |  |  | [-0:10, 0:13] | |  |  | [-0:09, 0:14] | |  |
| PC 3 | |  |  |  |  | -0:15 | * | -0.07% |  | -0:15 | * | -0.07% |  | -0:14 | * | -0.07% |
|  |  |  |  |  |  | [-0:29, -0:01] | |  |  | [-0:29, -0:01] | |  |  | [-0:29, 0:00] | |  |
| PC 4 | |  |  |  |  | 0:01 |  | 0.00% |  | 0:01 |  | 0.00% |  | 0:01 |  | 0.00% |
|  |  |  |  |  |  | [-0:06, 0:08] | |  |  | [-0:06, 0:08] | |  |  | [-0:05, 0:08] | |  |
| PC 5 | |  |  |  |  | -0:02 |  | -0.01% |  | -0:02 |  | -0.01% |  | -0:02 |  | -0.01% |
|  |  |  |  |  |  | [-0:06, 0:02] | |  |  | [-0:06, 0:02] | |  |  | [-0:06, 0:02] | |  |
| Age Group (39-45 ref.) | |  |  |  |  |  |  |  |  |  |  |  |  |  |  |  |
|  | Age 46-50 |  |  |  |  | -2:28 | *** | -0.61% |  | -2:34 | *** | -0.64% |  | -2:49 | *** | -0.71% |
|  |  |  |  |  |  | [-3:50, -1:05] | |  |  | [-3:56, -1:12] | |  |  | [-4:10, -1:27] | |  |
|  | Age 51-55 |  |  |  |  | -8:03 | *** | -2.02% |  | -8:16 | *** | -2.07% |  | -8:52 | *** | -2.23% |
|  |  |  |  |  |  | [-9:28, -6:39] | |  |  | [-9:41, -6:52] | |  |  | [-10:16, -7:28] | |  |
|  | Age 56-60 |  |  |  |  | -10:51 | *** | -2.67% |  | -11:10 | *** | -2.74% |  | -12:12 | *** | -3.02% |
|  |  |  |  |  |  | [-12:17, -9:25] | |  |  | [-12:36, -9:43] | |  |  | [-13:38, -10:46] | |  |
|  | Age 61-65 |  |  |  |  | -7:56 | *** | -1.98% |  | -8:14 | *** | -2.05% |  | -9:59 | *** | -2.50% |
|  |  |  |  |  |  | [-9:45, -6:07] | |  |  | [-10:05, -6:24] | |  |  | [-11:48, -8:10] | |  |
| Men | |  |  |  |  | -4:07 | *** | -0.94% |  | -4:27 | *** | -1.04% |  | -4:46 | *** | -1.15% |
|  |  |  |  |  |  | [-5:12, -3:02] | |  |  | [-5:34, -3:21] | |  |  | [-5:53, -3:38] | |  |
| Partner present | |  |  |  |  | 5:53 | *** | 1.57% |  | 5:28 | *** | 1.46% |  | 5:09 | *** | 1.37% |
|  |  |  |  |  |  | [4:38, 7:08] | |  |  | [4:12, 6:43] | |  |  | [3:54, 6:24] | |  |
| Child/grandchild present | |  |  |  |  | -2:13 | *** | -0.48% |  | -2:17 | *** | -0.50% |  | -2:31 | *** | -0.56% |
|  |  |  |  |  |  | [-3:15, -1:10] | |  |  | [-3:19, -1:14] | |  |  | [-3:33, -1:29] | |  |
| Years of Education | |  |  |  |  | 0:14 | *** | 0.07% |  | 0:13 | *** | 0.07% |  | 0:12 | *** | 0.06% |
|  |  |  |  |  |  | [0:07, 0:20] | |  |  | [0:07, 0:19] | |  |  | [0:05, 0:19] | |  |
| Urban | |  |  |  |  | -1:00 |  | -0.24% |  | -0:58 |  | -0.23% |  | -1:59 | ** | -0.47% |
|  |  |  |  |  |  | [-2:20, 0:20] | |  |  | [-2:18, 0:22] | |  |  | [-3:20, -0:39] | |  |
| Occupational Class (SIOPS) | |  |  |  |  | 0:00 |  | 0.00% |  | -0:01 |  | 0.00% |  | -0:01 |  | 0.00% |
|  |  |  |  |  |  | [-0:03, 0:02] | |  |  | [-0:03, 0:02] | |  |  | [-0:03, 0:02] | |  |
| Work hours (10-34 hours ref.) | |  |  |  |  |  |  |  |  |  |  |  |  |  |  |  |
|  | 35-44 work hours/wk |  |  |  |  | -10:11 | *** | -2.34% |  | -10:12 | *** | -2.35% |  | -9:13 | *** | -2.12% |
|  |  |  |  |  |  | [-11:24, -8:58] | |  |  | [-11:25, -8:59] | |  |  | [-10:26, -7:58] | |  |
|  | > than 44 work hours/wk |  |  |  |  | -18:36 | *** | -4.43% |  | -18:35 | *** | -4.42% |  | -17:31 | *** | -4.18% |
|  |  |  |  |  |  | [-20:07, -17:05] | |  |  | [-20:07, -17:04] | |  |  | [-19:03, -15:59] | |  |
| Manual Labour Job | |  |  |  |  | -0:55 |  | -0.32% |  | -0:50 |  | -0.29% |  | -0:42 |  | -0.26% |
|  |  |  |  |  |  | [-2:44, 0:55] | |  |  | [-2:40, 1:00] | |  |  | [-2:32, 1:07] | |  |
| Sedentary Job | |  |  |  |  | 0:20 |  | -0.02% |  | 0:26 |  | 0.01% |  | 0:12 |  | -0.04% |
|  |  |  |  |  |  | [-0:52, 1:33] | |  |  | [-0:47, 1:38] | |  |  | [-1:00, 1:24] | |  |
| Current Smoker | |  |  |  |  |  |  |  |  | -4:19 | *** | -1.11% |  | -4:07 | *** | -1.06% |
|  |  |  |  |  |  |  |  |  |  | [-6:13, -2:25] | |  |  | [-6:01, -2:13] | |  |
| Snores | |  |  |  |  |  |  |  |  | 1:54 | *** | 0.46% |  | 2:31 | *** | 0.62% |
|  |  |  |  |  |  |  |  |  |  | [0:52, 2:57] | |  |  | [1:28, 3:34] | |  |
| Alcohol Consumption (Never ref.) | |  |  |  |  |  |  |  |  |  |  |  |  |  |  |  |
|  | Special occasions |  |  |  |  |  |  |  |  | 1:48 |  | 0.61% |  | 2:05 |  | 0.68% |
|  |  |  |  |  |  |  |  |  |  | [-1:24, 5:00] | |  |  | [-1:06, 5:16] | |  |
|  | One to three times a month |  |  |  |  |  |  |  |  | 0:02 |  | 0.30% |  | 0:06 |  | 0.32% |
|  |  |  |  |  |  |  |  |  |  | [-3:03, 3:07] | |  |  | [-2:58, 3:11] | |  |
|  | Once or twice a week |  |  |  |  |  |  |  |  | 0:41 |  | 0.50% |  | 0:40 |  | 0.49% |
|  |  |  |  |  |  |  |  |  |  | [-2:07, 3:30] | |  |  | [-2:07, 3:28] | |  |
|  | Three or four times a week |  |  |  |  |  |  |  |  | 1:20 |  | 0.73% |  | 1:13 |  | 0.70% |
|  |  |  |  |  |  |  |  |  |  | [-1:29, 4:08] | |  |  | [-1:35, 4:00] | |  |
|  | Daily or almost daily |  |  |  |  |  |  |  |  | 0:44 |  | 0.49% |  | 0:50 |  | 0.51% |
|  |  |  |  |  |  |  |  |  |  | [-2:10, 3:37] | |  |  | [-2:02, 3:43] | |  |
| Weekly Commute (<= 15 miles/wk ref.) | |  |  |  |  |  |  |  |  |  |  |  |  |  |  |  |
|  | 16-40 miles/wk |  |  |  |  |  |  |  |  |  |  |  |  | -3:31 | *** | -0.80% |
|  |  |  |  |  |  |  |  |  |  |  |  |  |  | [-4:44, -2:17] | |  |
|  | > 40 miles/wk |  |  |  |  |  |  |  |  |  |  |  |  | -7:13 | *** | -1.70% |
|  |  |  |  |  |  |  |  |  |  |  |  |  |  | [-8:30, -5:55] | |  |
| Neuroticism Score (Std.) | |  |  |  |  |  |  |  |  |  |  |  |  | -4:22 | *** | -1.17% |
|  |  |  |  |  |  |  |  |  |  |  |  |  |  | [-4:53, -3:50] | |  |
| *N* | | 53211 | | |  | 53211 | | |  | 53211 | | |  | 53211 | | |
| *R^2^* | | 0.003 | | |  | 0.027 | | |  | 0.028 | | |  | 0.036 | | |

*Notes:* Beta coefficient (*B)* shows change in self-reported sleep duration (minutes:seconds). Confidence intervals, shown in parentheses, are calculated using bootstrapped standard errors (based on 1000 replications) that are clustered around the family ID. The ‘% Change’ column shows change in the percentage of total sleep duration; this is the *B* coefficient on the natural log of sleep duration. All estimates are produced from a one-third random sample of the UK Biobank, which includes individuals between the ages of 39-65 who are in paid employment of at least 10 hours per week. Nested models are shown which sequentially add covariates from variables from left to right. + p < 0.01, * p < 0.05, ** p < 0.01, *** p < 0.001

# Appendix G: Baseline Sensitivity Analyses Using Actigraphy-Derived Sleep Duration Measure

Table S10. *OLS Regression of Actigraphy-Derived Sleep Duration on Night Shift Work*

|  |  | 1 | | |  | 2 | | |  | 3 | | |  | 4 | | |
| --- | --- | --- | --- | --- | --- | --- | --- | --- | --- | --- | --- | --- | --- | --- | --- | --- |
|  |  | No Covariates | | |  | Demographics, Employment and Eve. PGS Adjusted for | | |  | Alcohol, Smoking and Snoring Adjusted for | | |  | Commuting and Neuroticism Adjusted for | | |
| Variable | | *B* | | *% Change* |  | *B* | | *% Change* |  | *B* | | *% Change* |  | *B* | | *% Change* |
| Regularity of Night Work (Never/rarely ref.) | |  |  |  |  |  |  |  |  |  |  |  |  |  |  |  |
|  | Sometimes works nights | -14:56 | ** | -5.37% |  | -10:10 | * | -3.93% |  | -10:10 | * | -3.92% |  | -9:49 | * | -3.83% |
|  |  | [-24:07, -5:46] | |  |  | [-19:21, -0:58] | |  |  | [-19:21, -0:58] | |  |  | [-18:59, -0:40] | |  |
|  | Usually works nights | -18:13 | * | -6.20% |  | -15:48 | + | -5.33% |  | -15:46 | + | -5.33% |  | -15:28 | + | -5.25% |
|  |  | [-36:19, -0:06] | |  |  | [-33:49, 2:13] | |  |  | [-33:45, 2:13] | |  |  | [-33:23, 2:27] | |  |
|  | Always works nights | -19:22 | * | -6.94% |  | -17:43 | * | -6.20% |  | -17:48 | * | -6.22% |  | -17:19 | * | -6.10% |
|  |  | [-34:15, -4:29] | |  |  | [-32:28, -2:57] | |  |  | [-32:34, -3:02] | |  |  | [-32:03, -2:36] | |  |
| *N* | | 12072 | | |  | 12072 | | |  | 12072 | | |  | 12072 | | |
| *R^2^* | | 0.002 | | |  | 0.047 | | |  | 0.048 | | |  | 0.050 | | |

*Notes:* Beta coefficient (*B*) shows change in accelerometer-derived sleep duration (minutes:seconds). Confidence intervals, shown in parentheses, are calculated using bootstrapped standard errors (based on 1000 replications) that are clustered around the family ID. The ‘% Change’ column shows change in the percentage of total sleep duration; this is the *B* coefficient on the natural log of sleep duration. All estimates are produced from a one-third random sample of the UK Biobank, which includes individuals between the ages of 39-65 who are in paid employment of at least 10 hours per week. Nested models are shown which sequentially add covariates from variables from left to right. + p < 0.01, * p < 0.05, ** p < 0.01, *** p < 0.001.

# Appendix H: Sensitivity analyses by sex

Since night shift work is more prevalent among males than females, we also performed additional analyses by sex. Table S11 shows the most common occupations involving night shift work by sex. For females, night work is overwhelmingly concentrated in nursing and personal care jobs, which together account for around 58% of all women’s night work. In contrast, for males we see that night shift work tends to be far more diffuse, with only around 21% concentrated in the top two occupations, protective services work and motor vehicle driving. In general, men’s night shift work tends to be concentrated in manual and skilled labour positions, whilst for women, these schedules are concentrated in service-oriented jobs.

Figure S6 shows sleep penalties by sex. While males in our sample on average sleep less, the sleep penalties associated with night work do not vary significantly with sex.

Figure S7 compares sleep penalties of night work in the male dominated jobs within our sample (craft and plant workers) to those penalties seen in female-dominated jobs within our sample (nursing and personal care). Whilst sleep duration is lower in male-dominated jobs, there are no systematic differences in the sleep penalties associated with night shift work between job groups.

Figures S8 and S9 show which occupations have the biggest sleep penalties associated with night shift work by sex. Note that ‘Sometimes’, ‘Usually’ and ‘Always’ night work have now been grouped to increase power. For both males and females, the largest sleep penalties are seen for models, salespersons and demonstrators. Corporate managers also experience significant sleep penalties for both males and females. For males, significant sleep penalties are also seen for machine operators as well as engineering professionals.

Table S11. *Most Common Occupations Involving Night Shift Work, by Sex*

| Females | | |  | Males | | |
| --- | --- | --- | --- | --- | --- | --- |
| Occupation | N | Pct. |  | Occupation | N | Pct. |
| Nursing and midwifery associate professionals | 578 | 34.5 |  | Protective services workers | 311 | 10.9 |
| Personal care and related workers | 387 | 23.1 |  | Motor-vehicle drivers | 292 | 10.3 |
| Protective services workers | 60 | 3.58 |  | Machinery mechanics and fitters | 170 | 5.98 |
| Health professionals (except nursing) | 58 | 3.46 |  | Messengers, porters, doorkeepers and related workers | 163 | 5.73 |
| Production and operations department managers | 49 | 2.92 |  | Production and operations department managers | 149 | 5.24 |
| Shop salespersons and demonstrators | 48 | 2.86 |  | building finishers and related trades workers | 113 | 3.97 |
| Client information clerks | 43 | 2.56 |  | Architects, engineers and related professionals | 102 | 3.59 |
| General managers | 42 | 2.5 |  | personal care and related workers | 96 | 3.38 |
| Travel attendants and related workers | 29 | 1.73 |  | Transport labourers and freight handlers | 72 | 2.53 |
| Optical and electronic equipment operators | 27 | 1.61 |  | Electrical and electronic equipment mechanics and fitter | 62 | 2.18 |
| Total | 1321 | 78.7 |  | Total | 1530 | 53.8 |

*Notes*: Categories shown are ISCO 3-digit. Night work includes those individuals who ‘Sometimes’, ‘Usually’ or ‘Always’ report working nights.

| Figure S6. *Effect of Night Work on Sleep Duration, by Sex* | Figure S7. *Effect of Night Work on Sleep Duration in Male- and Female-Dominated Occupations* |
| --- | --- |


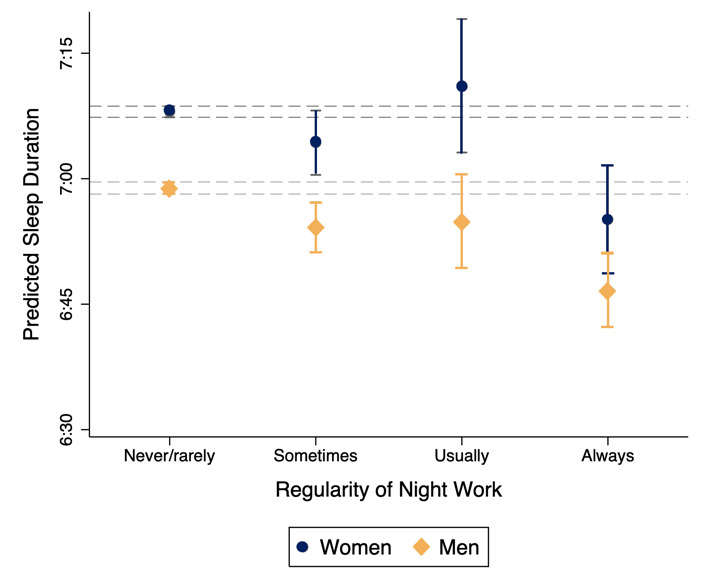

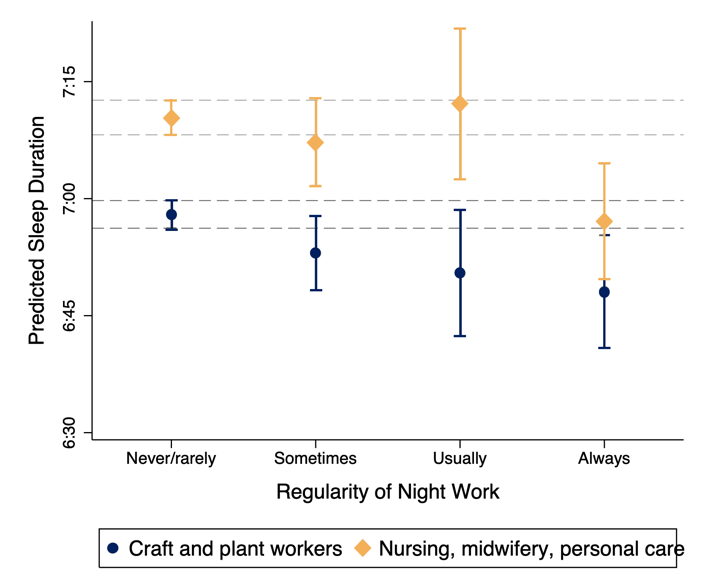


Figure S8. *Effects of Male’s Night Work on Sleep Duration, by Occupation*

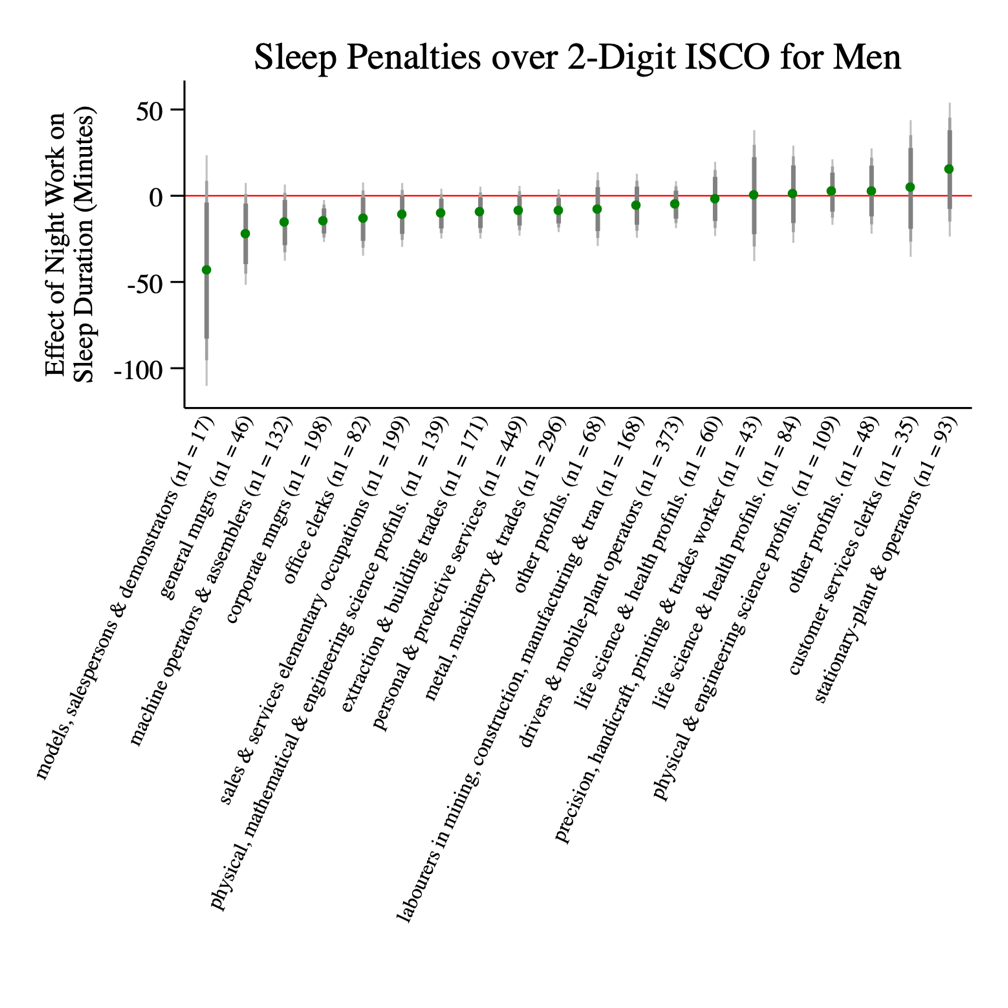


*Notes:* n1 = number of night shift workers. Occupations with less than 10 night workers or 30 workers total sampled have been omitted. ISCO refers to the International Standard Classification of Occupations.^13^

Figure S9. *Effects of Female’s Night Work on Sleep Duration, by Occupation*


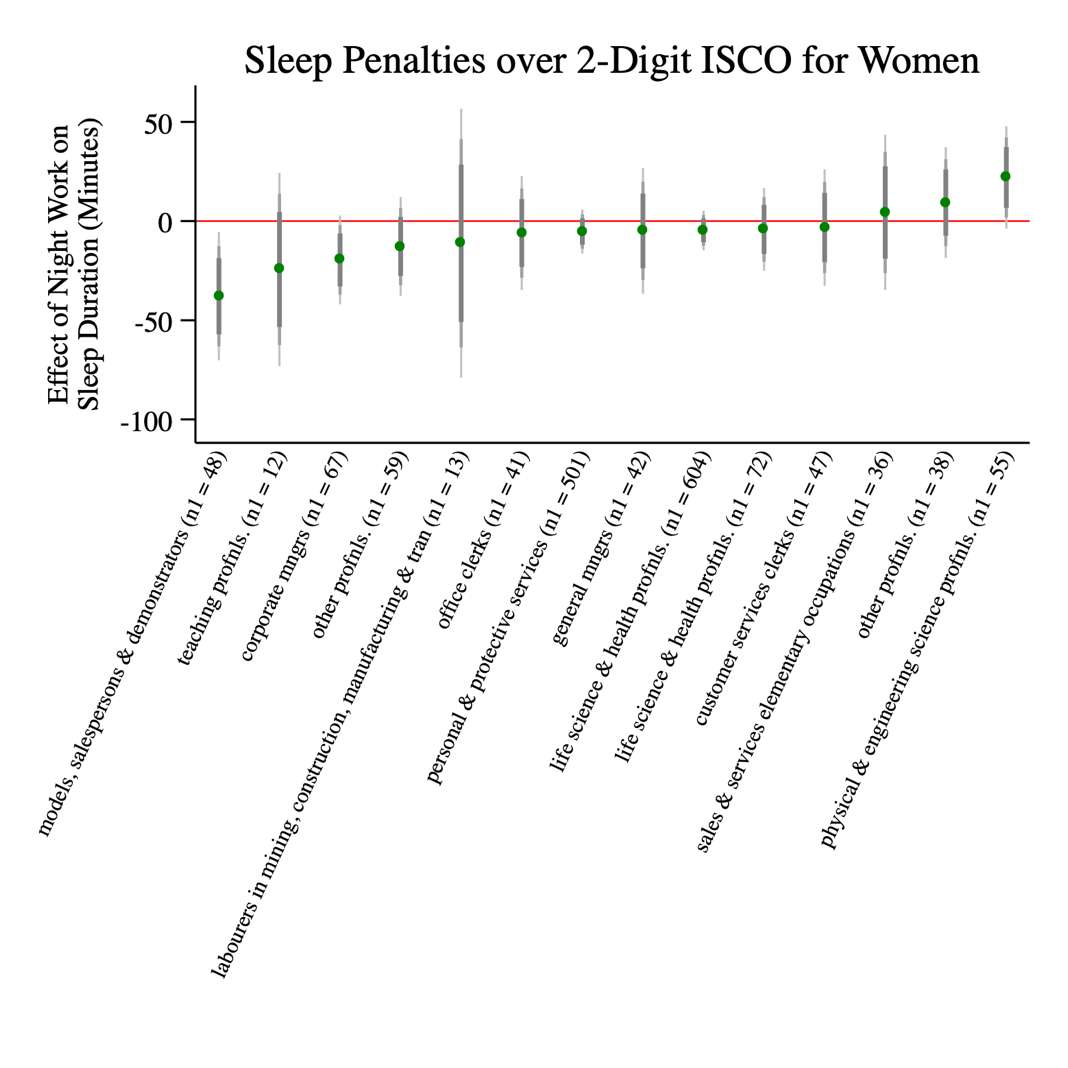


*Notes:* Occupations with less than 10 night workers or 30 workers total sampled have been omitted. ISCO refers to the International Standard Classification of Occupations.^13^

Table S12. *Linear Effect of Night Shift Work Interacted with PGS for Eveningness on Sleep Duration, over Sex*

|  |  | Females | | | | | |  | | Males | | | | | | |
| --- | --- | --- | --- | --- | --- | --- | --- | --- | --- | --- | --- | --- | --- | --- | --- | --- |
|  |  | 1 | |  | 2 | |  | | 3 | | |  | 4 | | |  |
|  |  | No Covariates | |  | All Covariates | |  | | No Covariates | | |  | All Covariates | | |  |
| Variable | | *B* | |  | *B* | |  | | *B* | | |  | *B* | | |  |
| Regularity of Night Work (Never/rarely ref.) | |  |  |  |  |  |  | |  | |  |  |  |  |  |  |
|  | Sometimes works nights | -4:12 | * |  | -3:71 | + |  | | -7:02 | | *** |  | -4:66 | * |  |  |
|  |  | [-8:02, -0:20] | |  | [-7:64, 0:21] | |  | | [-10:14, -3:90] | | |  | [-7:81, -1:52] | | |  |
|  | Usually works nights | 2:49 |  |  | 2:99 |  |  | | -5.05+ | |  |  | -3:95 |  |  |  |
|  |  | [-5:79, 10:78] | |  | [-5:14, 11:10] | |  | | [-10.81, 0:70] | | |  | [-9:51, 1:61] | | |  |
|  | Always works nights | -13:31 | *** |  | -13:02 | *** |  | | -14.22 | | *** |  | -12:22 | *** |  |  |
|  |  | [-20:12, -6:52] | |  | [-19:82, -6:22] | |  | | [-18.67, -9.77] | | |  | [-16:71, 7:74] | | |  |
| Eveningness PGS | | -0.06 |  |  | 0.16 |  |  | | -0.20 | |  |  | -0.20 |  |  |  |
|  |  | [-0.72, -0.60] | |  | [-0.49, 0.82] | |  | | [-0.92, 0.51] | | |  | [-0.90, 0.51] | | |  |
| Eveningness PGS * Night Work (Never/rarely ref.) | |  |  |  |  |  |  | |  | |  |  |  |  |  |  |
|  | Sometimes works nights | -0:06 |  |  | 0:35 |  |  | | 1:68 | |  |  | 1:52 |  |  |  |
|  |  | [-4:05, 3:94] | |  | [-4:30, 3:60] | |  | | [-1.27, 4.64] | | |  | [-1:41, 4:45] | | |  |
|  | Usually works nights | -1:78 |  |  | -1:60 |  |  | | 0:59 | |  |  | 0:76 |  |  |  |
|  |  | [-9:91, 5:34] | |  | [-8:52, 5:34] | |  | | [-4:91, 6:09] | | |  | [-4:56, 6:06] | | |  |
|  | Always works nights | 3:79 |  |  | 3:16 |  |  | | 4:40 | | * |  | 4:10 | + |  |  |
|  |  | [-2:44, 10:03] | |  | [-3:08, 9:40] | |  | | [0:08, 8:72] | | |  | [-0:22, 8:42] | | |  |
| *N* | | 27743 | |  | 27743 | |  | | 25468 | | |  | 25468 | | |  |
| *R^2^* | | 0.001 | |  | 0.037 | |  | | 0.003 | | |  | 0.027 | | |  |

*Notes:* Beta coefficient (*B*) shows change in accelerometer-derived sleep duration (minutes:seconds). Confidence intervals, shown in parentheses, are calculated using bootstrapped standard errors (based on 1000 replications) that are clustered around the family ID. All estimates are produced from a one-third random sample of the UK Biobank, which includes individuals between the ages of 39-65 who are in paid employment of at least 10 hours per week. Nested models are shown which sequentially add covariates from variables from left to right. + p < 0.01, * p < 0.05, ** p < 0.01, *** p < 0.001

# Appendix I: Moderating Effect of Eveningness PGS on Sometimes and Usual Night Work

| Figure S10. *Predicted Sleep Duration of Individuals Never/Rarely and Sometimes Working Nights, over PGS for Eveningness (Std.)* | Figure S11. *Predicted Sleep Duration of Individuals Never/Rarely and Usually Working Nights, over PGS for Eveningness (Std.)* |
| --- | --- |

**
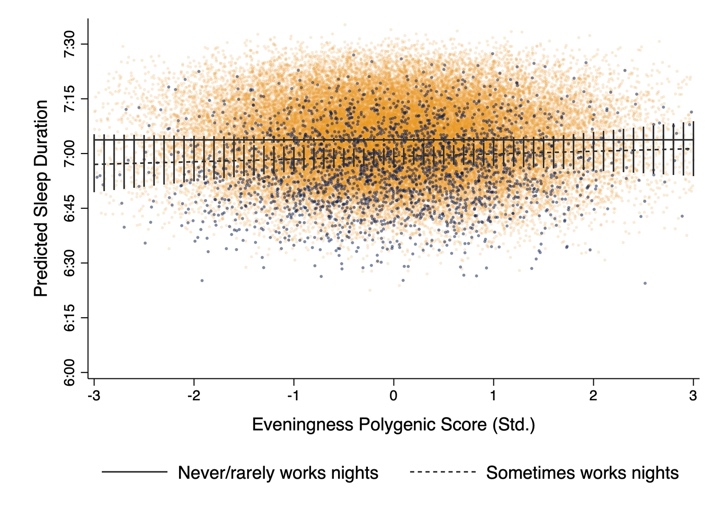

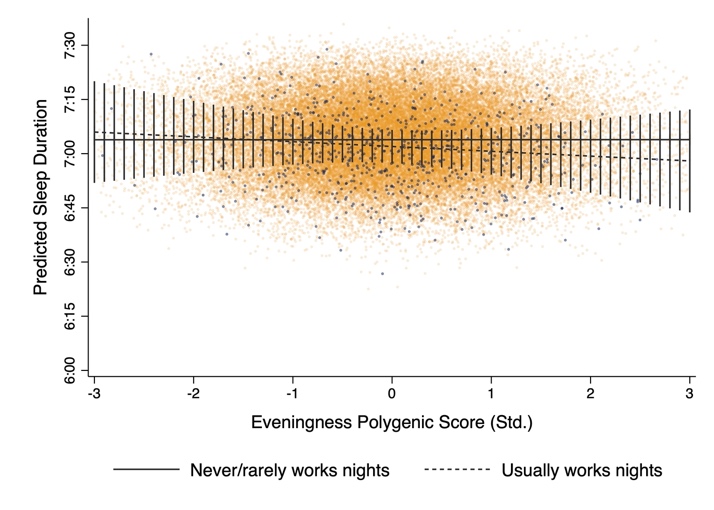
**

*Notes:* Estimates are conditional on the full set of covariates. Vertical capped lines show 95% confidence intervals, which are calculated using bootstrapped standard errors based on 1000 replications.

# Appendix J: Moderating Effect of Eveningness PGS on Always Night Work with Quadratic and Cubic Polynomials

| Figure S12. *Predicted Sleep Duration of Individuals Never/Rarely and Always Working Nights, over PGS for Eveningness (Std.) Squared* | Figure S13. *Predicted Sleep Duration of Individuals Never/Rarely and Always Working Nights, over PGS for Eveningness (Std.) Cubed* |
| --- | --- |

**
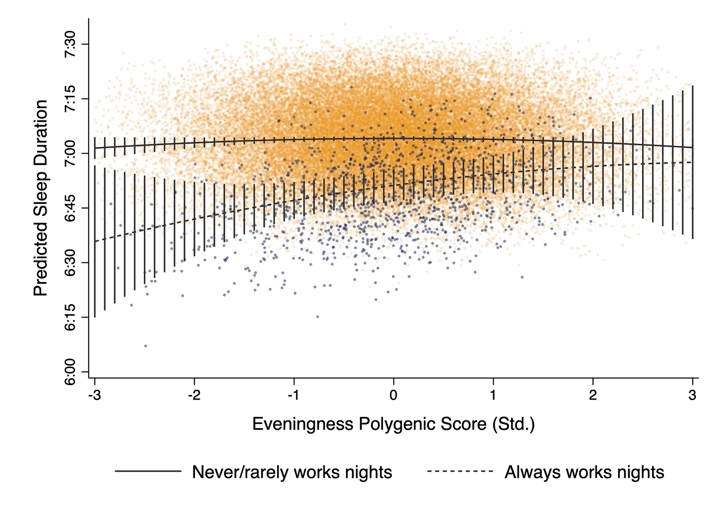

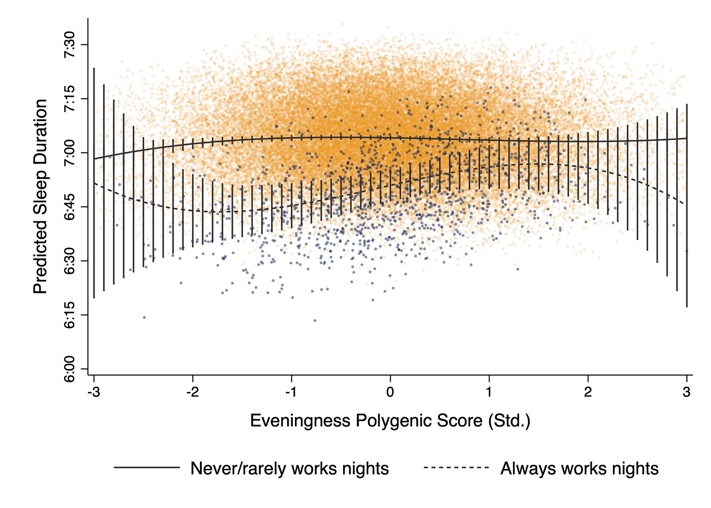
**

*Notes:* Estimates are conditional on the full set of covariates. Vertical capped lines show 95% confidence intervals, which are calculated using bootstrapped standard errors based on 1000 replications.

# Appendix K: Eveningness PGS Sensitivity Analyses Using Actigraphy-Derived Sleep Duration Measure

Table S13. *Linear Effect of Night Shift Work Interacted with PGS for Eveningness on Actigraphy-Derived Sleep Duration*

|  |  | 1 | | |  | 2 | | |  | 3 | | |  | 4 | | |
| --- | --- | --- | --- | --- | --- | --- | --- | --- | --- | --- | --- | --- | --- | --- | --- | --- |
|  |  | 5 PCs Adjusted for | | |  | Demographics and Employment Adjusted for | | |  | Alcohol, Smoking and Snoring Adjusted for | | |  | Commuting and Neuroticism Adjusted for | | |
| Variable | | *B* | | *% Change* |  | *B* | | *% Change* |  | *B* | | *% Change* |  | *B* | | *% Change* |
| Regularity of Night Work (Never/rarely ref.) | |  |  |  |  |  |  |  |  |  |  |  |  |  |  |  |
|  | Sometimes works nights | -15:08 | ** | -5.42% |  | -10:19 | * | -3.98% |  | -10:19 | * | -3.97% |  | -9:58 | * | -3.88% |
|  |  | [-24:19, -5:58] | |  |  | [-19:31, -1:07] | |  |  | [-19:31, -1:07] | |  |  | [-19:08, -0:48] | |  |
|  | Usually works nights | -18:19 | + | -6.21% |  | -15:28 | + | -5.23% |  | -15:24 | + | -5.22% |  | -15:07 |  | -5.14% |
|  |  | [-36:42, 0:05] | |  |  | [-33:43, 2:47] | |  |  | [-33:38, 2:50] | |  |  | [-33:18, 3:04] | |  |
|  | Always works nights | -19:15 | * | -6.90% |  | -17:44 | * | -6.21% |  | -17:50 | * | -6.23% |  | -17:21 | * | -6.11% |
|  |  | [-34:09, -4:21] | |  |  | [-32:32, -2:55] | |  |  | [-32:38, -3:01] | |  |  | [-32:08, -2:35] | |  |
| Eveningness PGS | | -2:01 | ** | -0.56% |  | -2:02 | ** | -0.57% |  | -2:00 | ** | -0.56% |  | -2:02 | ** | -0.57% |
|  |  | [-3:25, -0:37] | |  |  | [-3:26, -0:40] | |  |  | [-3:23, -0:37] | |  |  | [-3:25, -0:39] | |  |
| Eveningness PGS * Night Work (Never/rarely ref.) | |  |  |  |  |  |  |  |  |  |  |  |  |  |  |  |
|  | Sometimes works nights | -3:29 |  | -1.04% |  | -3:08 |  | -0.94% |  | -3:10 |  | -0.95% |  | -3:04 |  | -0.92% |
|  |  | [-12:33, 5:34] | |  |  | [-11:51, 5:35] | |  |  | [-11:53, 5:33] | |  |  | [-11:46, 5:39] | |  |
|  | Usually works nights | 4:29 |  | 1.23% |  | 3:05 |  | 0.91% |  | 3:19 |  | 1.00% |  | 3:13 |  | 0.97% |
|  |  | [-16:33, 25:30] | |  |  | [-18:17, 24:27] | |  |  | [-18:02, 24:39] | |  |  | [-18:04, 24:30] | |  |
|  | Always works nights | 6:32 |  | 1.93% |  | 4:22 |  | 1.35% |  | 4:39 |  | 1.43% |  | 4:51 |  | 1.48% |
|  |  | [-8:00, 21:04] | |  |  | [-9:27, 18:10] | |  |  | [-9:10, 18:28] | |  |  | [-8:56, 18:39] | |  |
| *N* | | 12072 | | |  | 12072 | | |  | 12072 | | |  | 12072 | | |
| *R^2^* | | 0.005 | | |  | 0.047 | | |  | 0.048 | | |  | 0.050 | | |

*Notes:* Beta coefficient (*B*) shows change in accelerometer-derived sleep duration (minutes:seconds). Confidence intervals, shown in parentheses, are calculated using bootstrapped standard errors (based on 1000 replications) that are clustered around the family ID. The ‘% Change’ column shows change in the percentage of total sleep duration; this is the *B* coefficient on the natural log of sleep duration. All estimates are produced from a one-third random sample of the UK Biobank, which includes individuals between the ages of 39-65 who are in paid employment of at least 10 hours per week. Nested models are shown which sequentially add covariates from variables from left to right. + p < 0.01, * p < 0.05, ** p < 0.01, *** p < 0.001.

Table S14. *Linear Effect of Night Shift Work Interacted with PGS for Eveningness on Actigraphy-Derived Sleep Duration, over Work Hours*

|  |  | 10-34 Hours/Week | | | | |  | 35-44 Hours/Week | | | | |  | 45+ Hours/Week | | | | |
| --- | --- | --- | --- | --- | --- | --- | --- | --- | --- | --- | --- | --- | --- | --- | --- | --- | --- | --- |
|  |  | 1 | |  | 2 | |  | 3 | |  | 4 | |  | 5 | |  | 6 | |
|  |  | No Covariates | |  | All Covariates | |  | No Covariates | |  | All Covariates | |  | No Covariates | |  | All Covariates | |
| Variable | | *B* | |  | *B* | |  | *B* | |  | *B* | |  | *B* | |  | *B* | |
| Regularity of Night Work (Never/rarely ref.) | |  |  |  |  |  |  |  |  |  |  |  |  |  |  |  |  |  |
|  | Sometimes works nights | -22:54 | ** |  | -22:23 | ** |  | -11:04 | * |  | -6:11 |  |  | -8:17 |  |  | -5:32 |  |
|  |  | [-38:27, -7:21] | |  | [-37:56, -6:51] | |  | [-21:50, -0:16] | |  | [-16:57, 4:35] | |  | [-22:26, 5:52] | |  | [-19:40, 8:36] | |
|  | Usually works nights | -31:50 | + |  | -31:25 | + |  | -15:59 |  |  | -14:40 |  |  | -5:42 |  |  | -4:35 |  |
|  |  | [-68:01, 4:21] | |  | [-67:24, 4:35] | |  | [-36:19, 4:21] | |  | [-34:50, 5:31] | |  | [-36:00, 24:36] | |  | [-34:48, 25:37] | |
|  | Always works nights | -10:30 |  |  | -13:31 |  |  | -16:58 | + |  | -15:05 |  |  | -20:56 | + |  | -24:34 | * |
|  |  | [-33:58, 12:57] | |  | [-37:05, 10:02] | |  | [-35:13, 1:16] | |  | [-33:10, 3:00] | |  | [-42:47, 0:54] | |  | [-46:47, -2:20] | |
| Eveningness PGS | | -3:23 | ** |  | -2:50 | * |  | -1:44 |  |  | -1:43 |  |  | -0:57 |  |  | -1:41 |  |
|  |  | [-5:50, -0:55] | |  | [-5:16, -0:23] | |  | [-3:51, 0:23] | |  | [-3:49, 0:22] | |  | [-4:10, 2:17] | |  | [-4:52, 1:29] | |
| Eveningness PGS * Night Work (Never/rarely ref.) | |  |  |  |  |  |  |  |  |  |  |  |  |  |  |  |  |  |
|  | Sometimes works nights | -6:40 |  |  | -6:30 |  |  | -9:47 | + |  | -7:53 |  |  | 6:38 |  |  | 6:52 |  |
|  |  | [-21:29, 8:08] | |  | [-21:12, 8:11] | |  | [-20:39, 1:05] | |  | [-18:35, 2:49] | |  | [-6:39, 19:55] | |  | [-6:13, 19:57] | |
|  | Usually works nights | -17:11 |  |  | -21:46 |  |  | 9:34 |  |  | 8:08 |  |  | 1:11 |  |  | -1:08 |  |
|  |  | [-66:10, 31:49] | |  | [-70:23, 26:51] | |  | [-12:56, 32:04] | |  | [-14:00, 30:17] | |  | [-31:01, 33:23] | |  | [-32:58, 30:41] | |
|  | Always works nights | 8:21 |  |  | 5:52 |  |  | 0:59 |  |  | 0:07 |  |  | 6:28 |  |  | 8:47 |  |
|  |  | [-16:30, 33:11] | |  | [-18:46, 30:31] | |  | [-17:47, 19:45] | |  | [-18:22, 18:34] | |  | [-14:26, 27:22] | |  | [-11:49, 29:23] | |
| *N* | | 3706 | |  | 3706 | |  | 5661 | |  | 5661 | |  | 2705 | |  | 2705 | |
| *R^2^* | | 0.010 | |  | 0.036 | |  | 0.004 | |  | 0.043 | |  | 0.005 | |  | 0.052 | |

*Notes:* Beta coefficient (*B*) shows change in accelerometer-derived sleep duration (minutes:seconds). Confidence intervals, shown in parentheses, are calculated using bootstrapped standard errors (based on 1000 replications) that are clustered around the family ID. All estimates are produced from a one-third random sample of the UK Biobank, which includes individuals between the ages of 39-65 who are in paid employment of at least 10 hours per week. Nested models are shown which sequentially add covariates from variables from left to right. + p < 0.01, * p < 0.05, ** p < 0.01, *** p < 0.001

# References

1. Mills MC, Barban N, Tropf FC. *An Introduction to Statistical Genetic Data Analysis*. Cambridge, MA: The MIT Press; 2020.

2. Manichaikul A, Mychaleckyj JC, Rich SS, Daly K, Sale M, Chen W-M. Robust relationship inference in genome-wide association studies. *Bioinformatics*. 2010;26(22):2867-2873. doi:10.1093/bioinformatics/btq559

3. Fry A, Littlejohns TJ, Sudlow C, et al. Comparison of Sociodemographic and Health-Related Characteristics of UK Biobank Participants With Those of the General Population. *Am J Epidemiol*. 2017;186(9):1026-1034. doi:10.1093/aje/kwx246

4. Jones SE, Tyrrell J, Wood AR, et al. Genome-wide association analyses in 128,266 individuals identifies new morningness and sleep duration loci. *PLoS Genet*. 2016;12(8). doi:10.1371/journal.pgen.1006125

5. Jones SE, Lane JM, Wood AR, et al. Genome-wide association analyses of chronotype in 697,828 individuals provides insights into circadian rhythms. *Nat Commun*. 2019;10(1):343. doi:10.1038/s41467-018-08259-7

6. Purcell SM, Neale B, Todd-Brown K, et al. PLINK: a tool set for whole-genome association and population-based linkage analyses. *Am J Hum Genet*. 2007;81(3):559-575.

7. Chang CC, Chow CC, Tellier LCAM, Vattikuti S, Purcell SM, Lee JJ. Second-generation PLINK: Rising to the challenge of larger and richer datasets. *Gigascience*. 2015. doi:10.1186/s13742-015-0047-8

8. Finucane HK, Bulik-Sullivan B, Gusev A, et al. Partitioning heritability by functional annotation using genome-wide association summary statistics. *Nat Genet*. 2015;47(11):1228-1235. doi:10.1038/ng.3404

9. Finucane HK et al. *Partionining Heritability by Functional Category Using GWAS Summary Statistics. BioRxiv*.; 2015. https://github.com/bulik/ldsc/wiki/Genetic-Correlation.

10. Euesdon J, Lewis C, O’Reilly P. PRSice: Polygenic risk score software. *Bioinformatics*. 2015;31(9):1466-1468. doi:10.1093/bioinformatics/btu848

11. Doherty A, Jackson D, Hammerla N, et al. Large Scale Population Assessment of Physical Activity Using Wrist Worn Accelerometers: The UK Biobank Study. Buchowski M, ed. *PLoS One*. 2017;12(2):e0169649. doi:10.1371/journal.pone.0169649

12. Willetts M, Hollowell S, Aslett L, Holmes C, Doherty A. Statistical machine learning of sleep and physical activity phenotypes from sensor data in 96,220 UK Biobank participants. *Sci Rep*. 2018;8(1):7961. doi:10.1038/s41598-018-26174-1

13. Ganzeboom HBG, De Graaf PM, Treiman DJ. A standard international socio-economic index of occupational status. *Soc Sci Res*. 1992;21(1):1-56. doi:10.1016/0049-089X(92)90017-B
